# Light Technology for Efficient and Effective Photodynamic Therapy: A Critical Review

**DOI:** 10.3390/cancers13143484

**Published:** 2021-07-13

**Authors:** José Francisco Algorri, Mario Ochoa, Pablo Roldán-Varona, Luís Rodríguez-Cobo, José Miguel López-Higuera

**Affiliations:** 1 Photonics Engineering Group, University of Cantabria, 39005 Santander, Spain; mario.ochoa@unican.es (M.O.); pablo.roldan@unican.es (P.R.-V.); lopezhjm@unican.es (J.M.L.-H.); 2 Instituto de Investigación Sanitaria Valdecilla (IDIVAL), 39011 Santander, Spain; 3 CIBER-bbn, Institute of Health Carlos III, 28029 Madrid, Spain; luis.rodriguez@unican.es

**Keywords:** photodynamic therapy, cancer, lasers, optical devices

## Abstract

**Simple Summary:**

Photodynamic therapy (PDT) is a promising treatment for cancerous tumours in which light technologies play a crucial role. An attempt to answer a long-standing question about which light source and light parameters are superior is carried out by reviewing the works reporting their effects on PDT outcome. We have identified the light characteristics that primarily affect the PDT process, based on the current evidence found in the literature. This review also examines cutting-edge technologies aiming to surpass the main challenge of PDT: low light penetration through the tissue. Whereas these technologies overcome several initial technical issues, they generate new challenges and pose limitations. We hope this review may be of interest to a broad audience, from bioengineers to clinical oncologists. The overall contribution we hope to make is to identify key roadblocks and provide a broad overview of light-based technologies, to foster improved developments and new perspectives towards enhanced PDT.

**Abstract:**

Photodynamic therapy (PDT) is a cancer treatment with strong potential over well-established standard therapies in certain cases. Non-ionising radiation, localisation, possible repeated treatments, and stimulation of immunological response are some of the main beneficial features of PDT. Despite the great potential, its application remains challenging. Limited light penetration depth, non-ideal photosensitisers, complex dosimetry, and complicated implementations in the clinic are some limiting factors hindering the extended use of PDT. To surpass actual technological paradigms, radically new sources, light-based devices, advanced photosensitisers, measurement devices, and innovative application strategies are under extensive investigation. The main aim of this review is to highlight the advantages/pitfalls, technical challenges and opportunities of PDT, with a focus on technologies for light activation of photosensitisers, such as light sources, delivery devices, and systems. In this vein, a broad overview of the current status of superficial, interstitial, and deep PDT modalities—and a critical review of light sources and their effects on the PDT process—are presented. Insight into the technical advancements and remaining challenges of optical sources and light devices is provided from a physical and bioengineering perspective.

## 1. Introduction

Cancer is currently one of the deadliest diseases causing millions of deaths every year. According to the world health organisation, cancer is the second leading cause of death globally, accounting for an estimated 9.6 million deaths, or one in six deaths, in 2018 [1]. Despite the established standard strategies (surgery, radiotherapy, and chemotherapy) have reasonable success for certain cancers, resistant cancer cells, recurrence, and metastases remain common. Cancer is adaptive and has some intriguing survival strategies. In some cases, it is capable of pumping out some drugs out of the cells or find alternative pathways to inhibit cell death. In early cancers, surgery can remove primary tumours, but undetected, residual cancer cells develop into a life-threatening recurrence. This versatility is one of the main obstacles to improving mortality rates. To do this, it is essential to provide further technologies for assisting and treating all cancer stages and, especially, to treat metastases. These new technologies would grant more chances of survival, including ineligible patients for standard management. However, these new technologies must be able to offer additional benefits or fewer side effects compared to the currently established ones. For this reason, finding alternative options is key for future cancer treatments. 

A non-conventional therapeutic modality for solid tumours, which offer advantages over standard treatments, is photodynamic therapy (PDT). In the current paradigm, the standard PDT relies on three main elements, a photosensitiser (PS), light, and molecular oxygen to elicit cell death through oxidative damage. First, a non-toxic PS is placed topically or injected systemically. After some time, the PS reaches a maximum concentration within the vasculature and subsequently in the tumour. When the PS reaches the maximum concentration at the tumour as compared to healthy tissue, appropriate light wavelengths excite the PS, which can transfer its excited-state energy, among other de-excitation processes, to either tissue substrate or surrounding oxygen. These reactions produce reactive oxygen species (ROS), specifically superoxide anion radicals and reactive singlet oxygen molecules, which kill tumour cells by both direct and indirect cell death mechanisms. Thus, the clinical effect can be produced by direct cell death (necrosis, apoptosis, among others), vascular damage (leading to tissue ischemia), immune modulation, or a combination of these. The efficacy of these mechanisms depends on many factors, such as the type of PS, cell, overall light dose or/and tumour oxygenation status, among others. PDT cytotoxicity mechanisms are different from the ones of chemotherapy, radiation therapy and immunotherapy (and their consequences too):

- PDT biological effects may be at least partially localised to the tumour, resulting in a higher concentration of the PS within the tumour in comparison to healthy cells.
- PDT uses non-ionizing radiation (in most cases) and its cytotoxic mechanisms produce limited damage to DNA and connective tissue structures (i.e., collagen), which after the treatment act as a scaffold enabling, potentially, the healing of the treated volume [2].
- Considering the previous point, this treatment could be used as many times as required by clinicians, something that is not possible with the current established treatments (surgery, chemotherapy, and radiotherapy). PDT has no “memory effect” as radiotherapy.
- There is also a rapidly increasing body of evidence that the damage and unique mechanism of PDT treatment on tumours and their microenvironments could inhibit drug resistance pathways and re-sensitize resistant cells to standard therapies [3].
- Emerging evidence now suggests that PDT can stimulate strong immunological responses, which depend on multiple factors that are being investigated [4]. This is a key effect to destroy tumours that extend to distant sites after local treatment and is actively investigated [5].

All of these different characteristics compared with those of the standard treatments, confer PDT an attractive option to be used alone or complementary (before or after) to current standard therapies. Despite the great potential of this technology, its application to deep-seated cancer and metastases remains challenging. Some issues must be solved to surpass actual technological paradigms. For example, limited light penetration depth, non-ideal photosensitisers, complex dosimetry, and complicated implementations in the clinic. Radically new light sources, advanced PS, measurement devices, and innovative application strategies are investigated to exploit the full potential of PDT. 

The general aims of this review are to highlight the advantages/pitfalls, technical challenges, and opportunities of PDT from a physical and engineering perspective with a focus on technologies for light activation of PSs such as light sources, delivery devices, and systems. Important stages for PDT such as detection, imaging, and dosimetry (monitoring and dose adaptation) to enable enhanced treatments, or clinical trials and cell death mechanisms, are beyond the scope of this review. For imaging, the reader is referred to [6,7] and for dosimetry to [8,9] (and references therein). If the reader is interested in cell death mechanisms, detailed reviews can be found elsewhere [10,11,12,13,14,15,16]. Finally, for a compilation of actual clinical trials, the interested reader is referred to [17]. We hope this review will be of interest to a broad audience, from bioengineers to clinical oncologists.

The manuscript is organised as follows. Section 2 is divided into three main subsections. First, a background subsection provides a brief description of the light absorption in tissues, PDT mechanisms and basic terminology of dose and beam parameters relevant for PDT. Then, we critically review the light sources and delivery devices for different PDT modalities. In this review, we include several PDT modalities into three main categories:

- Superficial PDT: involves skin treatments with low light penetration depth (typically <2 mm). It is also usually referred to as external PDT.
- Interstitial PDT (I-PDT): can treat tumours beyond 1 cm assisted by the use of needles, catheters, and optical fibres, but using conventional light sources—with its light penetration limits—similarly as superficial PDT.
- Deep PDT: includes a wide variety of technologies aiming at deeper penetration beyond what is achieved by conventional light sources. This section includes NIR radiation of upconversion materials, advanced PSs excited with novel nonlinear optical techniques, ionising radiation, self-illuminated compounds, and emerging implants.

In Section 2.2, conventional light sources and delivery devices for superficial and I-PDT are critically reviewed. Superficial and I-PDT are combined in one section because they share several common points such as the light sources type and some delivery devices (e.g., optical fibre technologies, among others). We provide another perspective on the role of the light sources (e.g., lasers, LEDs and broadband lamps) as the one typically found in previous reviews. For instance, in the first few years of the 2000s, a couple of reviews have described the capabilities and generalities of the current and emerging light sources for PDT at that time [18,19]. Recently, some others described the operating principles and basic physics of light sources highlighting advantages and disadvantages and reviewing the main studies using different light sources [8,20]. In previous reviews, less attention has been paid to the role of fundamental light characteristics (e.g., coherence and beam size) and light waveforms on the PDT efficacy and the light penetration into the tissue [21]. It is still debated if coherent or pulsed light is beneficial for PDT or light penetration. Thus, we provide a detailed and updated review focused on the impact of light coherence, beam size, and different light waveforms (e.g., pulsed and continuous wave) on PDT. Based on the existing literature, the main objective of this section is to highlight the evidence regarding the light source properties and/or parameters more suitable for PDT or deeper light penetration. 

Recent developments on deep PDT are addressed in Section 2.3. Alternative light sources for deep PDT include electromagnetic radiation such as NIR light using non-linear optic techniques (Section 2.3.1), ionising radiation such as X-rays or Cherenkov (Section 2.3.2), self-illuminated systems based on chemiluminescent and bioluminescent mechanisms (Section 2.3.3). Recently, another deep PDT modality that has gained attention due to advancements in nanotechnology and materials science is based on implants. The status and potential of emerging implants are critically reviewed in Section 2.3.4.

Then, we discuss the critical aspects of light sources for an efficient and effective PDT, and highlight the technical advancements and challenges of each PDT modality covered in this review.

## 2. Light Technology for PDT

### 2.1. Background

#### 2.1.1. Light Absorption in Biological Tissues

**Figure 1:**
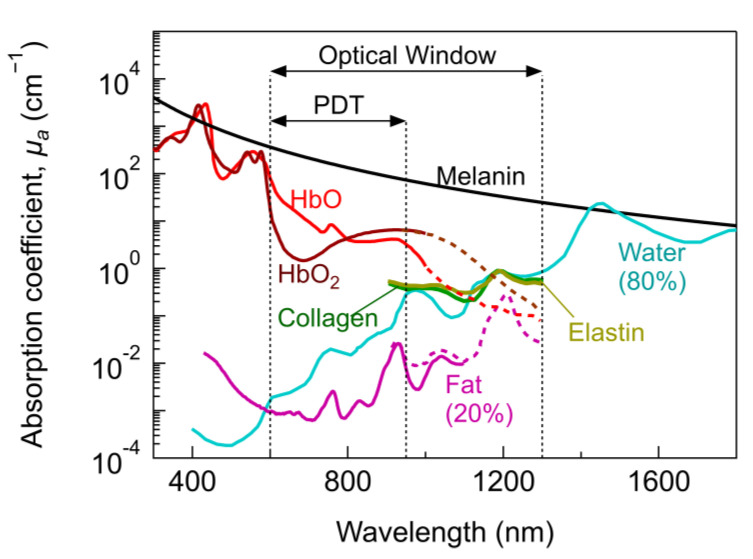
Absorption coefficient as a function of wavelength for several tissue constituents. Data for HbO (150 g/L), HbO_2_ (150 g/L) and melanin are taken from [23]. Purified pig and human (dashed lines) fat from [24] and [25], respectively. Water from [26]. Collagen and elastin from [25]. HbO_2_ and HbO shown as dashed lines are taken from [27] (adjusted to match the data at 1000 nm).

Being a light-based therapy, PDT strongly relies on the light–tissue interaction and its influence on the activation of the PS. Light–tissue interaction depends primarily on the light characteristics, absorption, and scattering properties of the biological tissue [18,19]. Tissues are composed of cells and elements with different sizes ranging from tenths of nanometers to tenths of micrometres and can be optically inhomogeneous depending on the tissue type [22]. Therefore, the optical properties, size, shape and density of these structures influence absorption and scattering in tissues. In general, the absorption properties of tissues can be separated into their tissue constituents. Figure 1 shows the absorption coefficient of primary tissue constituents such as haemoglobin, water, fat, elastin, collagen and melanin. Melanin and haemoglobin have the highest absorption coefficient ($\mu_{a}$) for wavelengths (λ) <1000 nm and dominate the absorption in this region. In spite $\mu_{a}$ of melanin being high, it is typically highly localized in low concentrations at specific regions (e.g., skin), thus its absorption is not relevant in most tissues. For λ > 1000 nm, water dominates the absorption with significant individual contributions from fat, collagen, elastin, etc., depending on the tissue type. Considering the $\mu_{a}$ of main tissue constituents, an optical window exhibiting the lowest light absorption lies between 600 to 1300 nm. More details on the absorption and scattering properties of specific tissues is given in Section 2.2.2.

Within the optical window, the so-called PDT therapeutic window is defined according to the optical properties and concentration of tissue constituents, the PS and the suitability for monitoring or imaging. Hence, defining unique therapeutic windows is difficult and several therapeutic windows with different ranges can be found in the literature [28,29,30]. On the one hand, PDT effects have been accomplished from UV to NIR, ranging from superficial to deeper tumour treatments. On the other hand, the most common PDT therapeutic window is usually restricted between ~600–950 nm (sometimes referred to as NIR-I) [29]. Superficial PDT lies within ~400–600 nm (UV–vis) [28]. For λ > 950 nm, water absorption increases significantly and may result in excessive tissue heating [29].

#### 2.1.2. PDT Mechanism of Action upon Absorption

PDT mechanism of action is based on the interaction of light, PS, and oxygen. The PS reacts with surrounding oxygen upon light excitation generating either free radicals (type I process) or singlet oxygen (type II process) capable to produce cell death mechanisms. The generation of singlet oxygen (^1^O_2_) is the most common process in PDT, and will be described below. Not all the competing photochemical and photophysical processes will be described, only those enabling a general—and simplified—description of the singlet oxygen generation in PDT. More details of such competing processes and energy transitions that may influence the PDT mechanism of action can be found in [31,32] and specifically about cell death pathways in [10,11,12,13,14,15,16].

**Figure 2:**
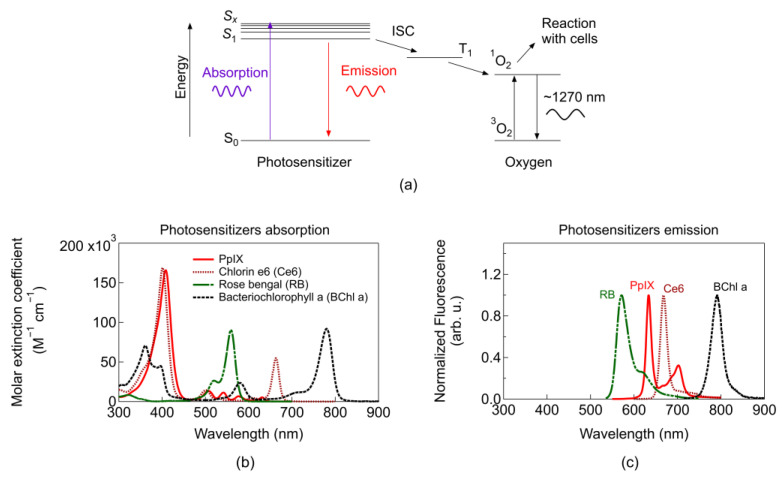
(**a**) Simplified Perrin–Jablonski diagram including PDT mechanism of action (ISC: Intersystem crossing). Each arrow indicates an energy conversion process (for simplicity not all possible conversions are depicted). Emission corresponds to the fluorescence of the PS and the 1270 nm emission from the singlet oxygen phosphorescence. (**b**) Molar extinction coefficient for absorption. (**c**) Fluorescence emission of typical PSs (data taken from [33,34]). Solvents used for measurements: chloroform for PpIX, toluene for Bacteriochlorophyll a, ethanol for rose bengal and chlorin e6. Additional parameters for common PSs, and PS targeting two-photon absorption can be found in pp. 32–36 in [7], [35], and [21], respectively.

Briefly, upon light excitation of the PS by absorption of suitable light wavelength, an orbital electron from the ground state (S_0_) is promoted to higher vibrational energy levels (Figure 2a). Such energy levels (S_x_) are unstable, therefore, after some time, (ps-regime) vibrational relaxation occurs (non-radiatively) and the electron may reach the lowest excited energy state (S_1_). In this state, it can undergo several possible de-excitation pathways. These pathways include the transition back to S_0_ non-radiatively, or radiatively emitting fluorescence (ns regime), or transition to a triplet state (T_1_) configuration (involving a flip of the spin of the electron) through a process known as intersystem crossing (ISC). Once in T_1_, the transition between T_1_ to S_0_ is spin-forbidden, thus T_1_ is a relatively long-lived intermediate state [7]. Since the ground state electronic configuration of diatomic oxygen is a triplet (^3^O_2_), it enhances the probability of energy transfer from the PS in T_1_ to surrounding oxygen [7]. Such energy transfer excites oxygen to its singlet state (^1^O_2_) and provides a mechanism for dissipation of the PS to return to the ground state [7]. The singlet reacts almost immediately with nearby cells and because the PS is not consumed in the process (unless photobleaching or photo-destruction of the PS occurs), the same PS could generate many singlet oxygen molecules. The direct energy transfer required for the transition from ^3^O_2_ to ^1^O_2_ is 0.974 eV (or 22.4 kcal/mol). However, T_1_ requires additional energy than 0.974 eV for the irreversible formation of ^1^O_2_, setting minimum energy of 1.13 eV, which is met by most PSs [7].

Most common PSs can be classified into three generations: (first) Photofrin based, (second) other single agents used alone or in combination [36], and (third) single agents coupled to molecules or nanoparticles that aid in localization, targeting, formulation, etc. [37]. In general, first-generation PSs exhibited long skin photosensitizing effects and suboptimal tissue penetration. Second generation PSs improved absorption within the optimal therapeutic window. Third generation PSs are mainly second-generation PSs bound to antibodies and liposomes for selective accumulation within the tumour tissue [36]. Figure 2b depicts the absorption coefficient of common PSs with absorption peaks lying within the energy range to fulfil the requirement for singlet oxygen generation. For imaging applications, blue light is typically used for monitoring PS fluorescence, which is employed as a contrast agent p. 15 in [7]. Photosensitiser fluorescence (Figure 2c) can be used to track tumours and is easier to filter or separate blue light from the emission light of the PS (~650 nm). Photosensitisers activated beyond 800 nm are usually not efficient in promoting an oxygen molecule from the triplet to the singlet state [8]; however, they are under continuous research and more details will be given in Section 2.3.1.

#### 2.1.3. Dose and Beam Parameters

The photodynamic dose is defined as the number of photons absorbed by the PS per gram of tissue [38]. In general, the properties of the PS (e.g., absorption coefficient, quantum yield, photobleaching rate), the local oxygen concentration in the treatment site, and the applied light determine the PDT dose. The PDT dose is very challenging to determine precisely [9]. Concerning light, the most important physical quantities are the fluence rate and time used for illumination because they are directly linked to the PDT dose. The radiant energy fluence rate is defined as the total power incident on an infinitesimal sphere and divided by the cross-section area of the sphere [38]. It has units of W/cm^2^. One could also define another parameter such as the fluence as the integral of the fluence rate over time with units in J/cm^2^. It is of paramount importance to report the fluence rate and the time used for illumination (or exposure time), and not only the fluence. The same energy fluence may be achieved by a light fluence rate of 1 W/cm^2^ for 1 s and 0.001 W/cm^2^ for 1000 s; however, each treatment may result in a very different PDT outcome. The reader is referred to [38] (and references therein) for more details regarding PDT terminology including dosimetry.

To define the fluence one should know the corresponding beam parameters. For continuous wave illumination (a light source emitting light continuously), the most important parameters are the power, centre wavelength, spectral bandwidth, beam spot size or cross-section and beam profile (e.g., Gaussian, top-flat). If a pulsed light is applied (a series of light pulses with on and off periods), additional parameters include the peak radiant power, average radiant power, frequency, and pulse width (or pulse duration on and off). 

**Figure 3:**
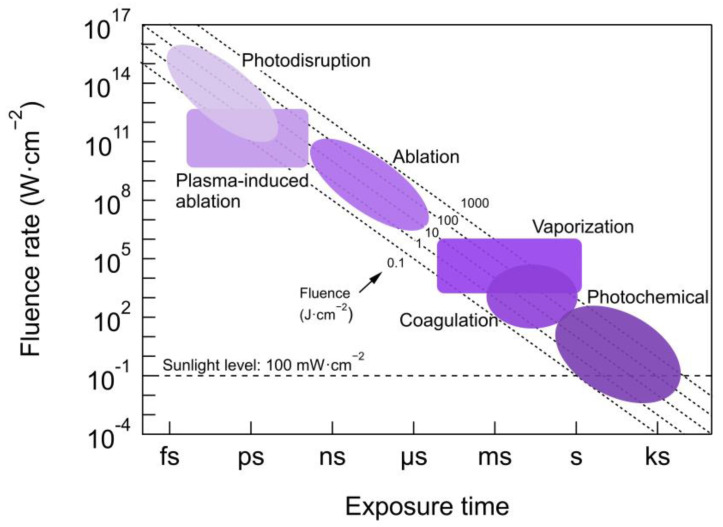
Different types of light–tissue interaction dependent on fluence and exposure times including PDT (within photochemical reactions region). Coagulation and vaporization are thermal effects caused by light exposure at a wavelength of high absorption in the tissue producing selective and localized heating. Ablation, involves the absorption of high energy photons (UV) exciting electrons to high non-bonding orbitals, breaking up the molecules and removing the tissue precisely without tissue damage arising from thermal effects. Plasma-induced ablation occurs when high fluence rates (>10^11^ W/cm^2^) of ns-pulses imping on the tissue and generate a plasma that precisely removes the tissue. Photodisruption includes plasma-induced ablation accompanied by mechanical processes such as acoustic and cavitation effects. More details about light–tissue interaction can be found in [40]. This figure is adapted from [40]. Photochemical reactions range use data taken from [40,42].

The influence on PDT efficacy of light source characteristics such as coherence, irradiated area or type of delivery (e.g., point, surface), etc., will be described in Section 2.2.1 and Section 2.2.2. Due to the strong implications of beam parameters and dose on PDT, a complete description of main guidelines and recommendations on how to properly report beam parameters, and other physical quantities has been provided by Jenkins and Carrol [39]. Figure 3 shows different types of light–tissue interaction for different exposure times and light fluence applied [40]. The most common fluences for PDT (covered by the shaded region corresponding to photochemical reactions) ranges from 0.1–200 J/cm^2^ and fluence rates below 300 mW/cm^2^ (in some cases tissue heating may occur for this upper limit) [8,41], which results in exposure times of seconds to tenths of minutes.

### 2.2. Conventional PDT: Superficial and Interstitial

The complexity underlying the guiding of light into the human body to activate PSs in deep target internal organs has traditionally made superficial PDT a main application of the technique. Most skin lesions are now treated by topical PS application, using ALA or MAL [43,44,45]. The topical application of PS allows only 1–2 mm of light penetration into the tissue, which is suitable only for treating superficial lesions [28]. ALA/MAL PDT has similar excellent tumour control rates for actinic keratosis and Bowen’s disease compared with surgical or topical chemotherapy [45,46]. Additionally, PDT is also approved in Europe for MAL treatment of superficial basal cell tumours of the skin, reporting a 95% response rate, that is, patients who had a complete response to therapy [28,47].

It is well-known light penetration is one of the main challenges for extended use of PDT [48,49]. For deep-seated or large tumours (>1 cm), I-PDT has emerged as a suitable type of treatment enabled by guiding light through optical fibres inserted into needles or catheters, and usually including a light delivery/dosimetry device [8,50,51]. The main drawback is that cancer has to be completely localized. Hence, I-PDT partially overcomes the deep penetration challenge by delivering light directly to the tumour. In addition, deep penetration achieved by the light source itself (optical fibres and catheters), and not by using complex delivery devices, is still beneficial to enhance treatment procedures and outcomes in many high-volume or thick tumours, since it may allow more homogenous extended illumination. The light source properties and parameters (e.g., coherence, wavelength, beam size, fluence, waveforms), delivery devices, the optical properties of the tissues, and PSs play a crucial role in light penetration into the tissue, as will be discussed below.

#### 2.2.1. Light Source Types

A wide variety of light sources (coherent and incoherent) have shown their capabilities to achieve PDT anti-tumour effects for different superficial [41] and interstitial treatment sites [8,18,49]. Thanks to the advent of laser sources, coherent light has been available for countless applications. Coherence is an ideal property of waves of equal frequency (monochromatic), which have a fixed relationship between phases. On the contrary, non-coherent waves have a random or changing phase relationship. Xenon lamps, metal halide lamps, lasers, and LEDs are some examples demonstrating beneficial PDT could be achieved independently of the light source characteristics [52]. The main advantages of coherent light sources against incoherent ones are their monochromaticity (can be targeted to narrow bands for specific PS), their high power, as well as their top-notch efficiency in the coupling of optical fibres (specifically for I-PDT). Besides, uniform irradiance can be easily achieved through iris and beam expanders [8,18]. It is possible to obtain precise control of the fluence that applies to the malignancy. Non-coherent sources stand out fundamentally for their low cost and by their wide illumination field (typically beam angles of around 120° [53]), because of their large beam divergence. This is highly advantageous for whole-body treatment, or superficial accessible tumours, e.g., skin or oral cavity [8], in which the need for additional optical elements is minimised. The required fluence rates in PDT are typically around 100–300 mW/cm^2^ [8] (for the upper limit and beyond tissue heating may occur). These values can be reached by non-coherent sources, therefore, the use of these types of sources does not necessarily involve an increase in the treatment time. Table 1 lists the fundamental characteristics of each type of source.

For I-PDT, solid-state lasers are the most commonly used light source, mainly because of the higher beam quality and better optical fibre alignment than other light sources (i.e., LEDs). I-PDT using lasers has been applied successfully to different treatment sites with localised tumours [49]. For instance, head and neck [54], pancreas [55], prostate [41,56], lung [57], brain [58], and more recently primary breast cancer [59,60]. In most cases, I-PDT has the main advantage to improve treatment outcomes for patients in which the standard of care therapy failed or needs better treatment options [49]. 

Alternatively, LEDs are promising light sources for cost-effective solutions and easier to transfer to clinical procedures for specific treatment sites and conditions. Nevertheless, LEDs have lower power output efficiency than lasers and given the broader spectrum, higher power is required to deliver equivalent fluence. Due to their lower electrical to optical conversion efficiency, thermal issues could be more important. All in all, the use of LEDs for PDT was demonstrated in the mid-1990s by Schmid et al. in brain tumours of canines treated with porifrin [52]. Presently, the rapid growth of LEDs led to the development of LED arrays of various wavelengths and handheld systems achieving the high power required for PDT [61,62,63,64]. For instance, array systems delivering a fluence rate of 100 mW/cm^2^ demonstrated a 90% of cell death in ovarian cancer cell line using LED modules reaching a fluence of 50 J/cm^2^ [62]. More recently, LEDs have been used for human breast cancer cell lines showing effective PDT without thermal issues [65] and for illumination of nanoparticles loaded with the photosensitizer in a human prostate tumour model [66]. As mentioned previously, thermal control is of paramount importance not only for avoiding possible damage to tissues and organs at risk but also because any temperature variation influences tissue optical properties and biological effects, adding complexity to dosimetry optimisation. Although LEDs could open pathways for an easier transfer to clinical procedures, just a few works have been reported targeting I-PDT applications. The ability of LED systems to achieve the power and thermal control requirements once coupled to fibre optics is still to be demonstrated. In contrast, for many superficial applications, LEDs are a simple and efficient solution widely accepted as a treatment light source.

Choosing one or another light source usually depends on the specific treatment site, cost, adaptability, wavelength—to activate PS—and power requirements for PDT. Several studies of coherent and incoherent light sources have been reported independently, using distinct parameters and conditions whose comparison is not possible [8]. Even though all possible parameters are kept equal, the comparison between LED and laser light involves additional complications [67]. For instance, laser light exhibit Gaussian beam profiles and typical parameters for the beam are usually not reported, e.g., beam cross-section [39]. Different beam cross-section would strongly impact the fluence applied and PDT outcome. Moreover, the impact of coherence on the light distribution within the tissue is still debated (as will be described in next section. Such impact would not take place using LEDs, or incoherent light. Overall, just a few works have reported a direct comparison between light sources, in which similar results [68,69] or slightly superior performance using lasers were found [70]. Therefore, it is not possible to conclude whether coherent or incoherent sources are more suitable for PDT. The main criteria followed on choosing the light source have likely remained practical, and to a lower extent fundamental. Undoubtedly, more efforts are required to demonstrate the type of light and its optimal characteristics for enhanced PDT.

#### 2.2.2. Penetration Depth and Light Source Characteristics

In PDT, besides the optical absorption of the tissue constituents (Section 2.1.1), the penetration depth is also limited by scattering mechanisms and light absorption of the PS. In general, the light penetration depth is proportional to an effective attenuation coefficient (including absorption and scattering properties) and depends logarithmically on the PS properties (concentration, absorption coefficient, quantum yield, and other effects such as saturation and photobleaching), and other factors—i.e., fluence rate, exposure time, etc. [71]. In this section, we focus on the role of the light characteristics affecting absorption and scattering events. For instance, light wavelength, phase (degree of spatial and temporal coherence), polarisation, and geometric characteristics such as the diameter of the spot size, spatial light patterns, among others [22]. In the following, only the light characteristics regarding wavelength, coherence, and spot size are covered. 

From diffusion theory and for wide uniform beams, the light penetration problem might be simplified to a useful and well-known analytical expression, i.e., $\varphi =kEe^{-\mu_{eff}z}$ [71]. The fluence rate is given by *φ*, E is the irradiance, *z* the depth of the tissue and *k* is a depth-dependent backscattering term usually ranging between 1 and 5 [22,71]. The effective attenuation coefficient ($\mu_{eff}$) is a function of the absorption ($\mu_{a}$) and scattering ($\mu_{s}$) components. The latter is further modified by an anisotropy factor *g* via $\mu_{s}^{\prime }$ = $\mu_{s}$(1 − *g*) that depends on the phase angle propagation of scattered light. The analytical expression might be an oversimplification of a complex problem of light–tissue interaction; however, it allows to highlight the main parameters affecting light propagation within tissues.

In this context, the attenuation of light in scattering media such as tissues (i.e., homogenous) is defined as $\mu_{eff}={\left(3\mu_{a}\left(\mu_{a}+\mu_{s}^{\prime }\right)\right)}^{1/2}$. To determine $\mu_{eff}$, both quantities, $\mu_{a}$ and the reduced scattering coefficient $\mu_{s}^{\prime }$, are wavelength-dependant measurable parameters [72]. Hence, the penetration depth is defined as $\delta \left(\lambda \right)=\mu_{eff}^{-1}\left(\lambda \right)$, and it is the distance in which light intensity decreases by 1/*e*.

**Figure 4:**
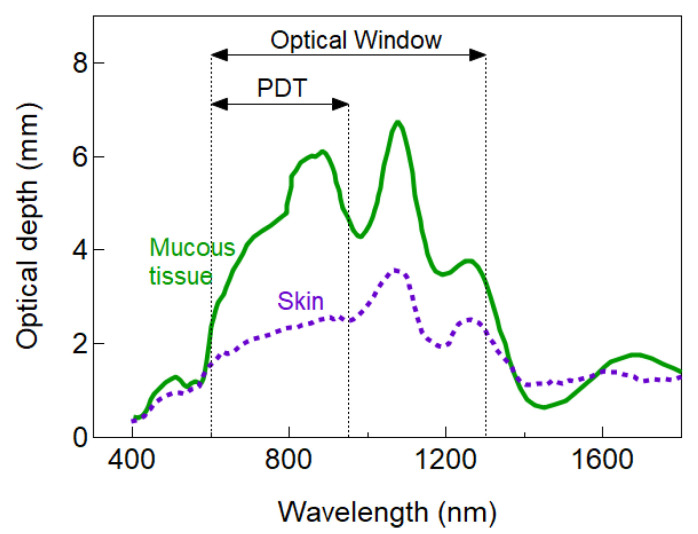
Example of the wavelength dependence of penetration depth for two different tissue types. Data were taken from [73]. The light wavelength has a strong impact on light penetration. For I-PDT, typically red light (around 650 nm) is employed and the expected penetration depth for most tissues is below 1 cm (<4 mm for mucous tissue).

Scattering is one of the main attenuators of light into the tissue. Main scatters lie within the range of the wavelength used for excitation of the PS, and even though photons are not lost due to scattering, it enhances the probability of absorption near the origin of the scattering event limiting the penetration depth. Scattering intensity is proportional to *λ^−^^w^*, where *w* depends on the characteristics and concentration of scatterers in the tissue. For particles much smaller than the wavelength, Rayleigh scattering dominates and *w* = 4. More realistic situations are *w* in the range of 0.2–1.7 [73]. Hence, longer excitation wavelengths are desirable because scattering intensity strongly diminishes with wavelength. Bashkatov et al. calculated the wavelength dependence of optical depth in different tissues [73]. Figure 4 shows an example of the wavelength dependence of δ for skin tissue and mucous tissue similar to that find in the bladder, stomach, oesophagus, among others. The maximum optical depth lies within the optical and PDT window, however, there are significant variations in optical depth even within the PDT window (>1 mm for mucous tissue). 

The optical penetration depth of Figure 4 is just an illustrative example to highlight the wavelength dependence of light attenuation that can be useful to select a suitable PS for a given tissue. However, such dependence might significantly differ from tissue to tissue. Indeed, higher complexity in the calculation of the penetration depth is also expected because the optical properties of living tissue may significantly vary depending on the content and concentration of tissue constituents and absorption of the PS. For instance, it has also been suggested cancerous sites (as compared to healthy cells) exhibit different optical properties caused by inflamed blood vessels (stronger absorption of haemoglobin) with δ about 7 times lower than normal tissue [74]. The complexity is even further increased because the optical properties of living tissue are also subject to patient-to-patient, site-to-site, and even temporal variations [75].

Besides wavelength, other light properties such as coherence may influence the penetration depth. Most tissues exhibit anisotropy factors *g* > 0.7 [75]. A positive *g* value implies that scattering is dominated by forward contributions; therefore, it is expected that coherent sources promoting forward scattering (i.e., laser) exhibit deeper light penetration. Within this context, the laser light interaction with tissue is often argued to modify the incident light, in which coherence and collimation are lost, due to scattering experienced after light propagation of a few micrometres [76]. Hence, it is reasonable to expect that any type of light (coherent or incoherent) would in principle act similarly inside the tissue, and the only desirable fact is that PS absorbs the incident light. However, this might not necessarily be the case. Fixler et al. found coherence is not lost for static tissue, but it is partially lost when there is a flow of fluid through the tissue, as suggested by the direct correlation of the volumetric flow with the loss of spatial coherence [77]. Hode et al. reported that laser light is still coherent enough to form laser speckles after passing through 2 cm thickness of highly scattering media (meat) [76]. The speckle pattern is not static due to fluid movement (in agreement with Fixler), but coherence is not lost within living tissue [76]. This means the speckle pattern fluctuates over time at a rate dependent on the fluid movement within the tissue but does not necessarily mean coherence is lost. If spatial coherence is not lost within the tissue, it may have additional implications. It was shown by Monte Carlo simulations that the speckle intensity distribution can exhibit individual regions with higher or lower fluence (up to 5 times) than the mean fluence [78]. An increased fluence at specific sites—within the proper limits to avoid tissue heating or damage—could achieve deeper regions with a sufficient threshold to activate the PS. A decreased fluence at specific sites could fall below the intensity thresholds required for PS activation. Hence, previous studies not only suggest an effective deeper penetration for coherent light sources could be achieved locally but also highlight that inhomogeneous light distribution could influence PDT mechanisms. Indeed, Rubinov has shown that laser light leading to speckle formation causes the appearance of inter- and intracellular gradient forces that affect biological processes [79]. To what extent these gradients and intensity variations impact PDT mechanisms (involving a PS) or light penetration is not well-known. Empirically, it has been difficult to compare and establish a deeper light penetration for either coherent or incoherent sources in experiments involving PDT effects. The few comparisons have been reported mainly on PDT efficacy terms (with similar results or without the possibility to benchmark between studies, see Section 2.1.1), which depend on other key parameters such as the structure of PS and localisation, drug-light time interval, treatment (oxygen) conditions, the fluence rate and time, etc. [80,81].

Alternatively, different effects can be found whether the light source causes a point treatment or a surface treatment, depending on whether the light locally strikes or totally covers the injured tissue, respectively. For PDT, the light at the activation wavelength of the PS is required, as well as the light fluence necessary to activate enough PS for lesion destruction. If this is accomplished for small (point treatment) or large (surface treatment) illumination areas, they can be used interchangeably. In this context, a wide illumination field is typically chosen to reduce the treatment time and achieve a greater penetration depth (as long as the irradiance remains constant) [82]. This has been shown using Monte Carlo simulations, in which light penetration increases with the beam size [82]. The penetration depth is approximately 10 times larger for the same fluence rate by increasing the beam diameter from 0.5 to 3 mm. Minimum increased penetration depth is estimated for beams larger than 5 to 10 mm, and no further increase above 10 mm (in agreement with [83]). Such simulations assume a flat beam, fixed anisotropy factor, absorption, and scattering coefficients for the two layers epidermis and dermis. According to [82], deeper penetrations in tissue due to wider beams are enabled by sufficient backscattering and a small value of µ_a_ compared to µ_s_. This combination of parameters is usually accomplished in most tissues [75]. Since the edge losses from the beam may extend three times the penetration depth, it is reasonable to reduce such losses by using beam cross-sections much greater than three times the penetration depth [75]. Hence, homogeneous light within the tumour is an important parameter not only to achieve a deeper penetration but also for nominally homogeneous PS activation. It should be reminded the previous reasoning applies for cases in which $\varphi =kEe^{-\mu z}$ effectively describes the tissue optics, and given the broad range of tissues and their heterogeneity found in some cases, this may no longer be valid and more accurate modelling is required [84].

Typically, large beam cross-sections (1–3 cm^2^) are required to treat large lesions such as those occurring in the skin [28]. In lasers, this is easily achieved using beam expanders. By adding spatial filters before the expander, it is possible to achieve a quasi-uniform illumination of the tissue [8,18]. For I-PDT, maximisation of tumour coverage is attempted by the use of different delivery devices along with advanced modelling (see Section 2.2.4).

The deeper light penetration is also pursued to a greater or lower extent following diverse approaches—namely, two-photon excitation [85], repeated PDT procedures [86,87], enhanced PS [88], a combination of PSs [89], X-rays, and internal bioluminescence [90]. The use of optical clearing agents is promising not only for deep-light delivery but also for characterisation and deep-monitoring [91,92]. More details on approaches related to Deep PDT will be given in Section 2.2.

#### 2.2.3. Pulsed, Continuous, and Other Light Waveforms for PDT

Continuous-wave (CW) and pulsed light are the most common waveforms used for superficial or I-PDT. Whereas CW sources are the workhorse for most PDT applications, just a few studies can be found regarding the effects of pulsed light on PDT. The main argument for beneficial PDT outcome using pulsed light is that light-off periods may allow tissue re-oxygenation and re-accumulation of specific PS at the lesion [21]. Such light-off periods could also allow the tissue to recover from a possible temperature rise. Regarding the light penetration, pulsed lasers achieving the same average fluence but using high fluence peaks could achieve a therapeutic threshold for PS activation in deeper regions of the tissue. This could be beneficial as long as a higher fluence peak rate does not increase the temperature to a point detrimental for the tissue.

Even though several studies reported the beneficial effects on PDT using either pulsed or CW waveforms, it is still debated if pulsed or CW exhibit deeper penetration depths or better PDT efficacy. In the following, a brief review of such studies is given.

Theoretically, Sterenborg et al. studied the effect of pulsed lasers on PS excitation and singlet oxygen yield [93]. The influence of the pulse duration and repetition frequency, and CW illumination on the PDT effect was also evaluated. They showed pulsed and CW have identical effects for rates below 40 kW/cm^2^. This value is far beyond the required fluence rate for PDT. At higher fluence rates, the effectiveness of PDT drops significantly. Then, Pogue et al. showed empirical slightly deeper penetration depths in tissue-simulating dosimeters by use of pulsed light (10-ns pulses of 10 Hz and maximum time-averaged irradiance of 300 mW/cm^2^) [94]. They suggested that consumption and photobleaching of the PS allow for less attenuation by the PS, and subsequent light can propagate deeper into the tissue. Kawauchi et al. reported on the effects of ns-pulsed light (1 MW/cm^2^ at 30 Hz and CW with a total light dose of 40 J/cm^2^) in photobleaching and oxygen consumption. They found, pulsed light promotes lower decomposition of PS and suppressed oxygen consumption, which resulted in a lower cytotoxicity effect compared to CW [95].

Recently, Grecco et al. used femtosecond laser pulses (temperature increase measured <4 °C) to compare two PSs. They found induced depth of necrosis with Photogen was greater with pulsed laser (2.0 ± 0.2 mm) compared with CW laser (1.0 ± 0.2 mm), whereas using Photodithazine induced greater necrosis with CW laser (2.9 ± 0.2 mm) compared with pulsed laser (2.0 ± 0.2 mm) [96,97]. They attributed higher absorption and saturation effects for the PS of second generation when pulsed light was used. This resulted in a lower generation of singlets able to promote cell death and highlighted the sensitivity of the PS to different light waveforms and PDT efficacy. Grecco et al. also reviewed in 2016 studies regarding pulsed and CW light sources (see [97] and references therein). The results are controversial and difficult to compare due to the different parameters and conditions used. The challenge for a proper comparison is to achieve the same fluence for a pulsed and CW source including the wide variety of pulsed time-regimes (from ns to fs) and keeping the other experimental parameters and conditions comparable. More recently, Klimenko et al. reported a theoretical analysis using pulsed light (200 ms pulse width and 700 ms repetition period). Singlet oxygen generation and tissue re-oxygenation benefited from a pulsed light source compared to CW [98]. They confirmed experimentally that the pulsed mode promoted apoptotic cell death of k562 cells in a pulsed mode, whereas CW induced necrotic cell death. Table 2 summarises the works using pulsed and CW found in the literature.

PDT effects using pulsed or CW have shown dependence with PS (as expected due to different absorption properties influencing light penetration depth and singlet oxygen generation), the light waveforms, and fluence applied. Photosentitiser saturation effects and photobleaching may also occur depending on the fluence rate, or peak fluence rate. Whereas saturation effects assist in achieving deeper penetrations (limiting PDT effect locally where PS is saturated), it could lead to photobleaching [94]. One common point among several studies is that PDT mechanisms using pulsed light are more favourable to induce apoptosis, whereas CW is prompt to induce necrosis [98,99,100]. Regarding light penetration, controversial results can be found either for interstitial or superficial applications [94,95,96,101].

The beneficial effect of pulsed or CW light for PDT is still an open question and many parameters have not been fully explored. Given the reported dependence of PDT efficacy on the fluence [80,81], it appears plausible to study the influence of different light waveforms on PS activation—considering different PS and oxygen concentration dynamics—and PDT of different types of light pulses and parameters, i.e., burst, super-pulse, pulse parameters (e.g., pulse width, shape). Less attention has been paid to advanced techniques such as wavefront shaping to achieve deeper light delivery penetration. Such techniques have demonstrated deeper light focusing, thus penetration depths. Besides, in certain cases (e.g., well-defined cancer margins), highly localized beams favour the selectivity of the treatment region with the potential to avoid organs at risk. Wavefront shaping can be accomplished by tailoring the multiple scattering light through the tissues varying spatial profiles and the phases of the incident wavefront [104,105,106]. This approach has been suggested mainly for enhancing penetration depth and optical resolution of optical microscopy techniques for imaging. Their higher complexity might not deem appropriate for light delivery in PDT. Hence, the feasibility of such techniques for deeper light delivery has to be envisaged.

#### 2.2.4. Delivery Devices

**Figure 5:**
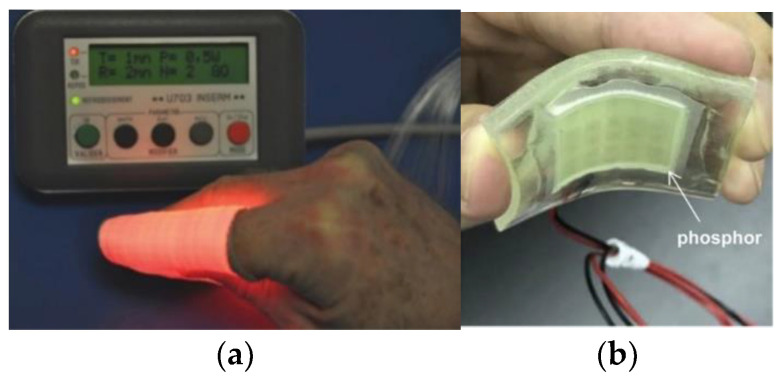
(**a**) Textile light diffuser applied on a finger-curved surface (image adapted from [112]). (**b**) Flexible LED unit designed for multi-wavelength excitation of 5-ALA (image adapted from [114]).

For superficial PDT, there are many novelties regarding delivery devices that have emerged recently. For instance, artificial daylight PDT is a field explored in recent times. Maire et al. conducted a study in 2020 whereby artificial white light sources were used as photoactivation alternatives to daylight for treating actinic keratosis [107]. This device (CE-marked) delivers uniform illumination with 2.9 mW/cm^2^ over a 314 cm^2^ surface. Likewise, different approaches have also been developed to obtain uniform light distributions for artificial daylight PDT, such as multi-wavelength LED [108], or non-coherent UV-protected greenhouses for therapy [109] and textile fabrics [110]. In 2018, O’Mahoney et al. implemented a uniform-illumination light source for artificial daylight photodynamic therapy [111]. The light source is capable of tuning the direction of light emission, thus providing uniformity across large anatomical surfaces, such as the head or leg. The light source implements LED chips that can independently emit seven distinct wavebands of light. In 2013, Cochrane et al. developed and tested a textile light diffuser based on commercial polymer optical fibre Figure 5a) [112]. The device is based on a wave pattern [113] and is designed to produce a large diffuser (useful width of 20 cm). The light source is a 5 W laser diode (635 nm) that generates a quasi-homogeneous intensity of 18.2 mW/cm^2^. Despite it is not very homogenous, it has the capability to tune the wavelength of the light source using the same diffuser and can be bent when applied to a non-planar surface. In 2019, Masuda et al. manufactured a flexible LED unit designed for multi-wavelength excitation of 5-ALA (Figure 5b) [114]. Therefore, it is possible to achieve more uniform irradiation of even areas, enhancing the therapeutic effects of PDT. Finally, because it is necessary to illuminate with light in therapy, it is equally important to have a real-time dosimetry system and, if possible, a probe for precise tumour delineation. Xie et al. developed a fibre optic probe for therapy guidance and monitoring [115]. It allows the tumour delineation using fluorescence/reflectance spectroscopy with an error of less than 5%.

**Figure 6:**
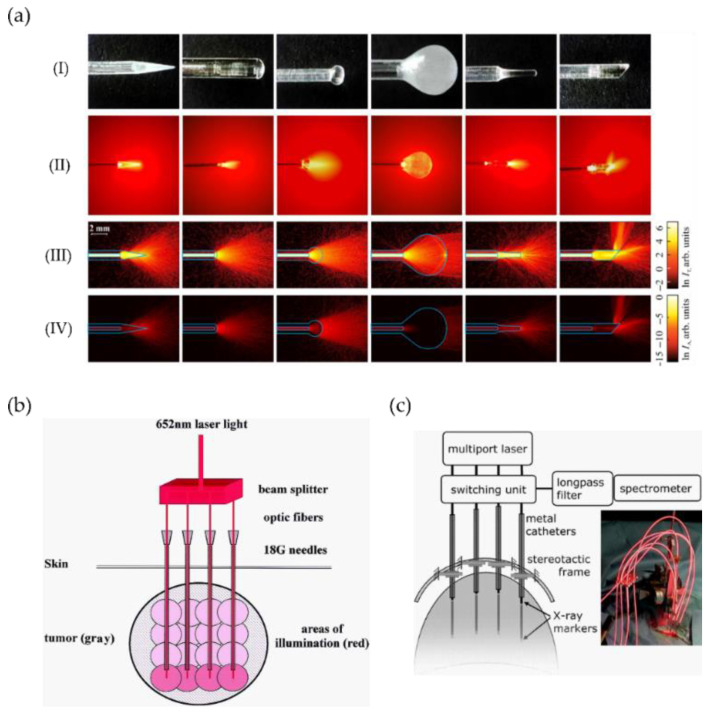
(**a**) Examples and application of advanced sapphire capillary needles for light delivery. (I) Sapphire tips; (II) laser light (632.8 nm) distribution produced by the sapphire tips in a diluted intralipid solution; corresponding to the (III) transmitted ln I_T_ and (IV) absorbed ln I_A_ intensities. The image and caption are adapted from their original created by Dolganova et al. [119] under a Creative Commons Attribution 4.0 Unsorted licence. (**b**) Schematic of I-PDT system including four fibres inserted into gauge-needles for simultaneous delivery light to treat head and neck cancer (image adapted from [121]). (**c**) Schematic of I-PDT system for brain tumour including real-time spectroscopic monitoring and clinical setting (optical fibres, catheters, and fixation device) during treatment (image adapted from [122]).

For I-PDT, delivery devices include diffusers of different geometries such as spherical, balloons, and cylindrical for wider illumination areas [8,116]. Their sizes range from some mm to tenths of mm and have been applied to several treatment sites—such as prostate, breast, oesophagus, lung, or biliary duct—among others. Other delivery devices such as fibre-focusing types have been benchmarked for oral surgery [117]. Recently, the use of sapphire capillary needles coupling optical fibres inside the needles with different tips and light patterns has been demonstrated [51,118,119]. Figure 6a depicts an example of such advanced capillary sapphire needles incorporating several types of needle tips with different geometry to control the direction of tissue light exposure and the amount of exposed tissue [119]. In this line, micro-focusing needles, allowing for lower energy required for treatment have also been developed. Such sapphire needles are advantageous because they can avoid degradation of their properties for multiple applications and sterilisation [120]. 

Complete systems including a set of fibres inserted into catheters or needles and guided through MRI (Figure 6b), X-ray markers (including real-time monitoring, see Figure 6c) or ultrasound images have been used for head and neck [121], brain [122], prostate [50], among others. For instance, the simultaneous light delivery using four fibres inserted into gauge needles and allowing for energy deposition of 20 J and an illumination area of about 10 mm in radius has been accomplished [121]. To achieve deeper penetration, the fibres are inserted into the deepest tumour area (guided by MRI) and subsequently withdrawn to illuminate other parts of the tumour (see Figure 6b). 

Similarly, one of the most advanced systems for I-PDT includes simultaneous dosimetry and light delivery. It has been applied to localised tumours in the prostate using a set of bare optical fibres—in principle, for more accurate measurement of optical properties—on a brachytherapy-type template [41,50,123]. The system can deliver light and monitor parameters simultaneously by use of a set of 18 fibres inserted into needles to deliver and monitor the excitation and light emission. Departing from an ultrasound input image, a 3D model of the prostate and tumour is generated and light dosimetry is constantly adjusted considering patient-to-patient variations in tissue optical properties.

Other delivery devices aiming at the maximisation of tumour coverage and increased PDT efficacy have been proposed, namely light blankets [124], optical surface applicators [125], scalpels combining ROS, oxygen, and light delivery [126], and novel light sources acting as optical batteries [127]. 

In previous studies, it has been widely demonstrated I-PDT efficacy is highly improved upon the implementation of optimised dosimetry and light delivery [8,9,50,128]. Dosimetry and light delivery are not challenging in superficial PDT because there are no organs at risk nearby, and it is possible to illuminate the full lesion [41]. In deep-seated tumours, besides proximity to organs at risk (OAR), there could be heterogeneity in tissue optical properties, PS distribution, and microenvironment before and during the treatment (tumour oxygenation and vascular network). Hence, real-time systems are crucial for the maximisation of I-PDT efficiency. Concerning the design of delivery devices, it is highly dependent on the treatment site and geometry, thus the delivery systems need to adapt to specific sites and incorporate monitoring stages for enhanced I-PDT. Investigations on the modelling and specific adaptable treatment planning considering various sites and optically heterogeneous tissues have been conducted to assist this purpose [84,129,130]. Despite proper dynamic dosimetry demonstrated its validity and success in specific cases, it is very challenging to achieve complete dosimetry, and many realistic implementations in the clinic may focus on determining the key factors governing dosimetry to perform the minimum essential measurements [9].

### 2.3. Deep PDT

This section addresses Deep PDT technologies aiming to overcome the challenge of low light penetration through the tissue from conventional light sources. Deep PDT sources are based on electromagnetic radiation such as NIR (Section 2.3.1), X-rays or Cherenkov radiation section (Section 2.3.2), self-illuminated systems enabled by chemiluminescent and bioluminescent mechanisms (Section 2.3.3), and novel implants positioned in—and in some cases within—the tumour microenvironment (Section 2.3.4).

#### 2.3.1. NIR Radiation

Within the PDT therapeutic window, a specific NIR window can be defined aiming for deeper light penetration through the tissues. It lies within 780 to 950 nm [29]. Despite this narrow window offers the highest transparency in tissues (Figure 1a and Figure 4), it has been limited by the lack of suitable PSs. Most common PSs are better excited in the visible region rather than NIR (see Figure 2b). Some approved NIR absorbing PSs are Talaporfin (664 nm) and Palladium bacteriopheophorbide (770 nm), and novel compounds with extended range are being investigated (a complete list can be found in [131]). 

In this context, there are several ways to enable the use of NIR radiation for PDT. In the following, we address three main approaches to excite directly or indirectly the PSs: (i) two-photon absorption, (ii) novel nonlinear optical photon conversion techniques, and (iii) the use of the so-called upconversion materials and nanoparticles (UCNPs). Any of these approaches involve the upconversion of two or more photons to higher energy levels to directly (i) or indirectly (ii) excite the PS by subsequent radiative light emission or non-radiative energy transfer (iii).

Two-photon absorption (TPA) is a nonlinear optical process involving simultaneous absorption of two NIR photons that combined promote an electron to a higher energy level than a single photon. TPA excites the PSs directly, or indirectly by exciting dedicated nanomaterials as will be described below. For direct excitation, specifically designed PSs are required [85,132]. To enable direct excitation of the PS through TPA, light sources with ultra-fast pulses (<10 ps) of high photon density (focused beams) are typically required due to the low absorption probability of the TPA process in most PSs. Advantageously, TPA is a nonlinear process and the absorption increases quadratically with the laser intensity enabling the excitation of the PSs [132]. One main advantage of TPA is the use of laser-focused beams, localising the illumination area, thus providing high selectivity (preserving organs at risk in well-defined cancer margins) and minimizing off-target toxicity [21]. Another advantage is that PS conjugates for TPA show very little autofluorescence in the biological window, which is beneficial for optical imaging [133]. In the first attempts using TPA for PDT, the technique demonstrated the capabilities to excite some common PSs; however, it was not enough to promote cytotoxic effects [134]. Hence, the low TPA cross-section of common PSs (e.g., PpIX, Photofrin, or Visudyne) was identified as the main bottleneck on the use of TPA [21]. Then, the development of new PSs enabled the demonstration of TPA with deep light penetration (about 2 cm) and its effectivity in vivo [29], and in a living mammal when applied to blood vessels occlusion [135]. TPA typically requires high fluence rates and not easily accessible systems, and despite the increased efficiency of the two-photon absorption cross-section, the low anti-tumour effects still limit its use.

Recently, a radically new approach was proposed by Kachynski et al. [136]. It consists of the indirect excitation of the PS (also using ultra-fast high-intensity lasers) by exploiting nonlinear optical photon conversion mechanisms occurring in many biological tissue constituents. These mechanisms include second-harmonic generation (SHG), and four-wave mixing (FWM), including coherent anti-Stokes Raman scattering (CARS). As stated by Kachynski et al., SHG is a second-order nonlinear optical process occurring in collagen, which is abundant in tumours and CARS/FWM, a third-order nonlinear optical process produced by proteins, lipids, nucleic acids, and aquatic biological environments [136]. Kachynski et al. demonstrated deeper light penetration and phototoxicity effects with lower radiation thresholds by using a combination of these novel techniques as compared to TPA alone. Fluences employed ranged between 30–90 J/cm^2^ (no significant thermally induced cytotoxicity was detected by control experiments of irradiated samples without PS). After 75 scans (4500 J/cm^2^), nearly 70% of cells were necrotic or detached by employing SHG/TPA to excite chlorin e6, which was higher as compared to CARS/FWM/TPA or TPA alone. They suggested that many reported TPA induced PDT studies using lasers in the range of 750–850 nm may have contributions from the SHG signal (at ~400 nm) caused in fibrillar collagens of tumours. Finally, they concluded SHG by collagen fibrils contributes efficiently to the excitation of chlorin e6 producing photodamage.

Another indirect excitation of the PSs is based on the upconversion of NIR photons into visible photons using dedicated upconversion materials, such as nano-transducers or upconverting nanoparticles (UCNPs) [137,138,139,140,141,142,143]. Nano-transducers potential candidates are gold nanorods (several orders of magnitude higher two-photon cross-sections than other PSs for TPA) [144], semiconductor quantum dots (QDs) [145] or carbon-quantum dots to avoid toxic elements from QDs [146]. Concerning UCNPs, they are made of ceramic lattice host doped with rare-earth ions (lanthanides) [21]. They can be adjustable in size, shape, and light emission through rational design and suitable doping [147]. One of the main advantages is the use of NIR radiation achieving deeper penetration, and the light emission in the visible region in which most PSs are better excited (Table 3). Besides, the power density required to excite the UCNPs is significantly lower than TPA, i.e., 1–10^3^ W/cm^2^ and 10^6^–10^9^ W/cm^2^, respectively [21]. Hence, besides TPA, CW NIR-excitation enables the absorption of two photons. To allow UCNPs emission, the energy from a NIR-excited fluorescent molecule (donor) is transferred to a second molecule (acceptor), which in turn emits the desired wavelength. This energy transfer can be accomplished by a non-radiative process known as Förster resonance energy transfer (FRET). To enable FRET, proximity between the chromophores or fluorochromes is required (50–100 Å because efficiency is inversely proportional to the sixth power of the distance between donor and acceptor) [148,149], and the absorption spectrum of the acceptor chromophore must overlap the fluorescence emission spectrum of the donor [150]. We will not provide further details because excellent recent reviews exist for this topic [90]. However, we can highlight some of the main challenges are the retention difficulty of the UCNPs within the microvasculature system, and the potential toxicity caused to the normal cells by current upconversion materials [151,152].

#### 2.3.2. Ionising Radiation

The ionising radiation consists of electromagnetic waves or subatomic particles, with enough energy to ionize atoms or molecules by detaching electrons from them. The energy from one ionisation can break chemical bonds in molecules causing damage to the living tissue. In the case of electromagnetic waves, only those with very high frequencies can be considered as ionising—e.g., gamma rays, X-rays, and the higher ultraviolet part of the electromagnetic spectrum. For subatomic particles, there are four kinds of ionising radiation, namely Beta (consisting of electrons),Alpha (two protons and two neutrons), protons and neutrons. The sources can be natural (any radioactive materials) or artificial (nuclear reactors, particle accelerators, and X-ray tubes).

**Figure 7:**
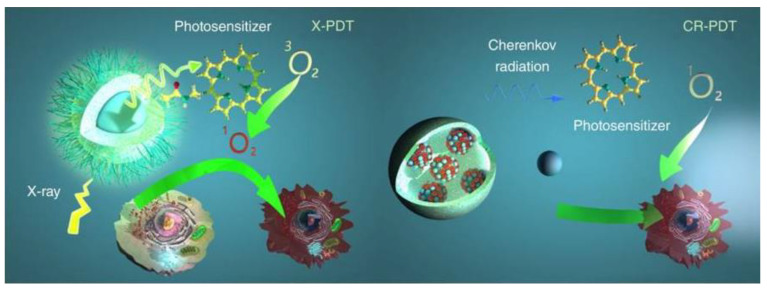
Schematic illustration for X-PDT and CR-PDT. Left: Classic X-PDT. X-rays excite a nanoscintillator to generate X-ray luminescence, which in turn activate a PS to produce cytotoxic ROS. Right: Cherenkov radiation PDT. Cherenkov radiation from radioisotopes is harnessed to activate a PS to initiate PDT. Reproduced with permission from B. Cline et al. Wiley Interdisciplinary Reviews. Nanomedicine and Nanobiotechnology published by Wiley, 2019 [179].

Ionising radiation has been proposed as an alternative source for PDT achieving deep tissue penetration, specifically, X-ray and Cherenkov radiation (see Figure 7).

On the one hand, the required X-ray doses in clinical radiotherapy are in the range of hundreds of keV to MeV, making it difficult to be used with traditional PS. To overcome this problem, in 2006 the use of X-rays and nanoparticles was proposed [180]. Since then, numerous studies have been reported, which are collected on recent reviews [179,181,182,183,184]. In the case of X-ray PDT, the PS can be activated by either persistent luminescence nanoparticles (PLNPs) or scintillating nanoparticles (SCNPs). With PLNPs the X-ray energy is stored at the defects or electron traps, causing a long-lasting afterglow which continuously serves as a light source for PDT activation (can emit light from few minutes to several days) [185]. SCNPs down-convert X-rays energy into visible light through a scintillation process and then transfer the energy to nearby PS. Some reported studies can be found in Table 4. They can be classified into two major groups, namely doped scintillator and semiconductor [179]. Some important characteristics are a high material density (for a good ionising radiation interaction), high scintillation quantum yield and efficient energy transfer as well as biocompatibility, and adapted in vivo bio-distribution [186]. In the case of a doped scintillator, one of the most studied have been lanthanides with high material density, high atomic number, and strong luminescence intensity [187]. In the case of semiconductor SCNPs the size has to be small to maximize quantum entanglement effects [179]. Despite the promising results, most of the X-ray PDT studies were mainly obtained with cancer cell lines or animal models bearing subcutaneous grafted cancer cells, thus limiting clinical relevance [186].

On the other hand, Cherenkov radiation (CR) was proposed in 2011 as an alternative source for in vivo photoactivation [203]. This option can be used along with radiotherapy or even without any external radiation (by using radioactive isotopes) [203]. As it is defined in [186], Cherenkov light is a luminescence signal produced by charged particles that travel faster than the phase velocity of light in a dielectric medium (it can be compared with sound barrier crossing, but for light). Cherenkov photons are produced by successive polarization/depolarization of the medium along the particle path, yielding constructive interferences. When a radioactive isotope decays, charged particles such as Beta are generated. As these particles travel through the dielectric molecules, they polarize the molecules and while returning to their ground state, the molecules emit the energy as photons. If the particles have a speed lower than light in that medium, the polarization field around the moving particle is usually symmetric, so the corresponding wavefronts do not interfere. On the contrary, if the particles have a speed higher than light in that medium, the molecules do not have time to go to the ground state before the particle has left, resulting in an asymmetric polarization field, that causes a constructive interference of the wavefronts with a cone-like light emission (at a specific angle). The spectrum of radiation luminescence consists of continuous wavelengths throughout the ultraviolet and visible spectrum, where the number of photons per wavelength is proportional to 1/λ^2^ [204].

Some studies have demonstrated that it is possible to use CR from radionuclides to activate oxygen-independent nanoPSs, such as titanium dioxide (TiO_2_) [205,206] and aminolevulinic acid (ALA) [207]. However, there are some doubts about Cherenkov action in free radical production and cell death. There is also a previous study reporting activation of TiO_2_ nanoparticles from radioactive ^32^P without invoking Cherenkov luminescence as the mechanism of action [208]. The main problem is the extremely low fluence rates of Cherenkov radiation, as an example, the average Cherenkov emission from ^18^F is approximately three photons per radioactive decay in water (refractive index *n* = 1.33) over the 250–800 nm range [208]. Monte Carlo simulations have determined the flux rates for radionuclides to be on the order of 0.01–1 nW/cm^2^ [209], several orders of magnitude below some reported in vitro and in vivo fluence rates required for PDT, which have found a decrease in cytotoxicity for fluence rates below 5.5 mW/cm^2^ [210]. These results indicate that the implied mechanisms are not completely understood. Another issue is the radiation caused by some radioisotopes. For example, one standard radiotracer used in positron emission tomography (PET) has an effective radiation dosage of 5–8 mSv [211]. To compare with X-ray radiation, some common procedures such as chest X-ray or chest computer tomography (CT) have a radiation dosage of around 0.1 mSv [212] and 8 mSv [213], respectively. An approximate comparable time of natural background radiation exposure for a chest X-ray is 10 days and for a chest CT around 2 years [214]. Moreover, CR has proven very inefficient, it needs high doses of charged particles, it gives broad wavelength radiation up to a limit, and it tends to be concentrated in the UV and blue light (as commented before, these wavelengths does not have much penetration depth). Despite this, Cherenkov radiation has potential advantages, for example, the suppression of external irradiation sources or the selective accumulation of many radiopharmaceuticals in tumours after systematic injection (targeting multiple metastases) [215].

#### 2.3.3. Self-Illuminated Systems

Self-illuminated systems have emerged as a promising solution to limited penetration depth without ionising sources. There are mainly two mechanisms to produce light without an external source, Chemiluminescence Resonance Energy Transfer (CRET) and Bioluminescence Resonance Energy Transfer (BRET) [216]. As their names indicate, Chemoluminescence is the emission of light as the result of a chemical reaction whereas Bioluminescence is produced by a living organism. In fact, Bioluminescence is a form of chemiluminescence that occurs in some animals and microorganisms. CRET involves a chemiluminescent (CL) donor whose energy is transferred to a suitable biological acceptor (oxidizing agent) and then, the adjacent PS is activated. Since the first proposal of luminol intracellular CL in 2002 [217], this has been the most used excitation source in CRET PDT with an emission peak wavelength of 425 nm [218,219,220]. One advantage is that luminol can be mixed with hydrogen peroxide (H_2_O_2_), abundant in the tumour microenvironment, to activate the PS. The main drawback is the limitation on PS selection due to the fixed emission peak of these systems. Some works have used also RET intermediate systems to tune the emission peak, as semiconducting polymer dots [221], conjugated polymer nanoparticles (which can luminesce and supply oxygen) [222] or carbon dots [223]. The peak emission can be matched to the peak absorption of the PS. However, one problem of using RET intermediate systems is the reduction of PDT efficiency (as several energy transfer steps are required before ROS generation). Despite this, some works have reported toxicities of 90% on SMMC-7721 cancer cells (using Ce6 PS) [223]. Despite these reported systems meet the spectral overlap requirement for CRET, and have high cytotoxicity and considerable tumour inhibition, there are biocompatibility issues such as low specificity and toxicity for healthy cells.

Alternatively, BRET PDT consists of a light-emitting molecule and a catalysing enzyme (usually called luciferin and luciferase, respectively) that in combination produce bioluminescence (BL). Some commonly reported sources are Firefly and Renilla. In the first case, Firefly luciferase (fLuc) is the most important and studied BL system [224]. This system depends on the surrounding pH with a peak wavelength of 560 nm and 620 nm for basic and acidic pH, respectively [225,226]. The first report of the use of this BL for PDT was made in 2003 by Theodossiou et al. [227]. The authors reported ^1^O_2_ was produced because of the combination of BL with hypericin PS (a 90% of toxicity rate was measured). A few years later, a similar study demonstrated that firefly BL emission is not sufficient to generate a cytotoxic effect because of insufficient photon generation [228]. These results are intriguing, and highlight the necessity of questioning if the cytotoxic effects reported can be attributed to the BL emission only, or additional factors are implied.

**Figure 8:**
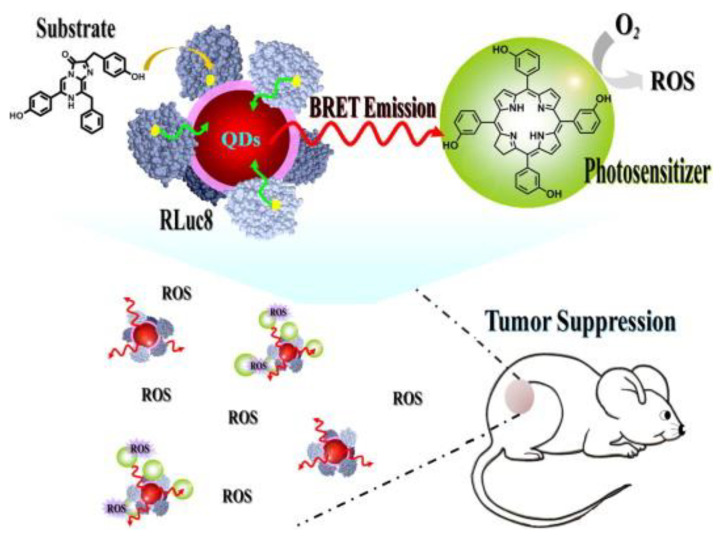
Schematic representation of RLuc8-immobilized QDs-655 for BRET-based PDT. Reproduced with permission from C.-Y. Hsu et al., Biomaterials; published by Elsevier, 2013 [231].

It is known that self-luminescence is relatively weak due to the difficulty to have the substrate and the catalyser close and relatively low quantum yields (that depends on the surrounding medium pH). To increase the quantum yield, recent works have proposed the use of RET intermediate systems. In 2019, K. Yang et al. used carbon nanodots (CDs) to upconvert the fLuc emission (to 440 nm). The CDs were functionalized to the PS (PpIX, absorption peak around 400 nm) that in contact with the fLuc system produced significant ROS in vitro [229]. In the case of Renilla luciferase, when combined with coelenterazine luciferin a blue-green peak wavelength of 480 nm is produced [230]. This system has been tested as an intracellular excitation source for PDT by Hsu et al. [231]. They took advantage of the quantum dots (QDs) emission at 655 nm to activate the PS (m-THPC, Foscan) because it is easier to tune the emission of a quantum dot than the BL system (Figure 8).

Other similar systems have used other PS as chlorin e6 (Ce6) [232]. Despite a delay in tumour growth (higher when using QDs than without them) and localization on the external surface of cells were demonstrated, there was significant toxicity to healthy cells. Besides, the authors have demonstrated that BRET energy generates a stronger PDT effect in the cellular membrane than a 1000× higher laser energy dose and the efficiency is considerably higher than firefly BL PDT [228,231]. However, several shortcomings must be addressed, e.g., other components different from quantum dot must be explored (due to toxicity), the two-step energy transfer efficiency has to be increased, the BL and PS have to be in the same subcellular location (selective delivery) and the used PS has to be optimized for the emission wavelengths. To overcome these problems, recent works have proposed gene transfection using luciferase-fused genes (some risk of genotoxicity) [233] and BL-induced proteinaceous PDT based on a protein biosensor, capable of generating ROS without the need of PS [234].

In conclusion, the potential of self-illuminated systems is enormous but they are still in their infancy. Several reports using CL and BL have demonstrated intracellular activation of different PS. Among them, Rluc–coelenterazine and luminol systems have been able to induce considerable PDT effect both in vivo and in vitro. Despite this, it cannot be found in the literature a plausible explanation about discrepancies of generated light fluences found in different articles. The main cause could be the required short distance between the substrate, the catalyst and the PS (and the variability of this parameter in real systems). Further research on advanced delivery systems, which can locate these components efficiently to the cancer region at the same time, is required. In this regard, if this is achieved, CL/BL systems will maintain one of the main advantages of PDT, its selectivity (reducing also the cytotoxicity to healthy cells). On the other hand, it has to be considered that this kind of systems depends on various energy transfer steps, which can decrease the overall PDT effect. Hence, unravelling these processes is key towards a better understanding of self-illuminated systems.

#### 2.3.4. Implants

The miniaturization of electronics is opening pathways for the fabrication of implants of various sizes (mm-range or less) and shapes suitable to attach to several treatment sites. The implants consist of light sources encapsulated into a bio-compatible polymeric material [235]—with high optical transparency—that can be implanted on the tumour microenvironment, and externally activated by an electromagnetic radiation source. Several light sources have been incorporated inside the implants to activate the PS, namely: (1) persistent luminescent materials (so-called “optical batteries”) such as nanoparticles (PLNPs) or green persistent luminescence materials (GPM), (2) UNCPs, and (3) micro-devices including LED sources and modules. For activation of the light sources, NIR radiation [236,237,238], radio-frequency (RF), near-field communication (NFC) [239,240,241], and ultrasound [242] have demonstrated their capabilities to activate the light sources.

One of the main benefits of the use of implants is the possibility to perform metronomic PDT (mPDT) [243]. This is a modality in which low light dose (typically <1 mW/cm^2^) and slow or repeated infusion of PS is pursued over extended periods (from some hours up to tenths of hours). The use of low fluence rate over extended periods has shown clinical benefits over conventional PDT fluence rates (typically ~100 mW/cm^2^) [81,210,240,244,245,246]. For instance, it was found that repeated PDT yielded necrosis at deeper regions than typically expected for conventional PDT [246]. Other benefits from the use of mPDT are PS photobleaching prevention and lower oxygen consumption in the tumour due to the low light fluence rate applied, which in turn avoid any potential thermal issues. Fluence rates over 75 mW/cm^2^ lead to fast oxygen consumption that may not be resupplied by the blood flow through the tumour vasculature [80]. In the following, we describe recent selected studies that offer a broad overview of the general status of emerging implants for PDT.

**Figure 9:**
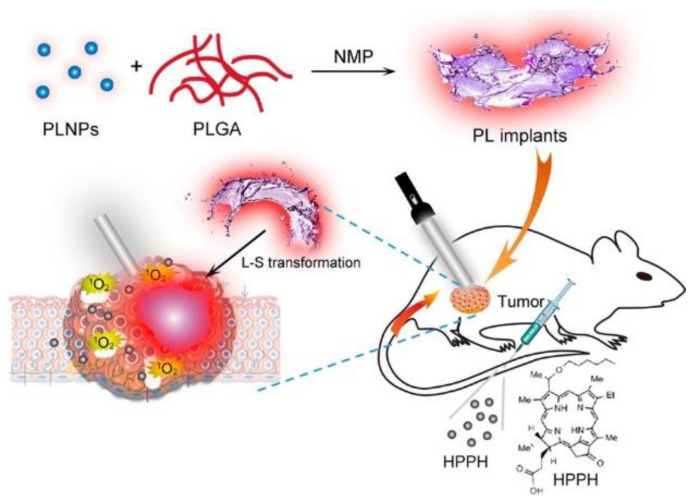
Schematic illustration of the construction of ZGC PL implants for in vivo LED/PersL–PDT upon LED irradiation. Reproduced with permission from W. Fan et al., ACS Nano; published by American Chemical Society, 2017 [236].

Fan et al. designed an injectable material that can produce persistent luminescence after NIR irradiation [236]. The implants consist on a dissolution of ZnGa_1.996_O_4_:Cr_0.004_ (ZGC) PLNPs in poly(lactic-co-glycolic acid) (PLGA)/N-methylpyrrolidone (NMP) oleosol (see Figure 9). After the oleosol injection, the material turns into a solid attaching firmly to the tumour region (despite Cr is a heavy toxic element, no biocompatibility issues have been observed). The ZGC shows an enhanced afterglow intensity (peak at 695 nm) during hours after a short activation of a few minutes. The used PS was 2-(1-hexyloxyethyl)-2-devinyl pyropheophorbide-α (HPPH, Photochlor). The authors demonstrate a remarkable tumour reduction in the first week, and a complete suppression in about half a month (with an additional dose of PS at day 7 and LED irradiation 24 h later).

In [237], L. Hu et al. used green persistent luminescence materials (GPM), which can store energy from UV-blue light, added to PS Rose Bengal (RB) and mixed with upconversion phosphorous. For PS excitation, the NIR radiation of 980 nm is upconverted to 520 nm through a combination of upconversion materials (NaYF4:25%Yb,0.5%Tm) and UV rechargeable persistent phosphors (SrAl_2_O_4_:2%Eu^2+^, 4%Dy^3+^). No intravenous (IV) injection of upconversion materials is required avoiding possible toxicity problems. All components are mixed with CaO_2_ (to overcome hypoxia tumour) and added to polydimethylsiloxane (PDMS). The PDMS is finally solidified at 60° for 1 h. The device is implanted on a 100 mm^3^ tumour. Applying 2 W/cm^2^ for 5 s, the luminescence can persist for more than 2 h (it has to be noted that the authors do not take into account maximum power exposure limits to 980 nm excitation for human skin [247]). Despite the ^1^O_2_ is generated inside the PDMS, the authors reported the singlet oxygen can diffuse through the PDMS reaching the tissue, and achieving cytotoxic effects. Both in vitro and in vivo results showed inhibition of tumour proliferation.

Another NIR-activated implant relays on UCNPs (NaYF_4_:Yb^3+^, Er^3+^ and NaYF_4_:Yb^3+^, Tm^3+^) mixed with poly(ethylene glycol) diacrylate (PEGDA) and a fluorinated ethylene propylene (FEP) cladding layer to improve light transmission and minimize leakage of UCNPs [238]. The PS (5-ALA) is administrated systemically. The implant is activated with an irradiance of 725 mW/cm^2^ of NIR at 980 nm (within the limits of 726 mW/cm^2^ of maximum permissible exposure for human skin). A brain tumour is studied, the results reveal beneficial outcomes and biocompatibility of the implant in comparison to interstitial fibre optics. Optical fibre produced higher glial scarring comprising both reactive microglia and astrocytes as compared to UCNPs implant [238]. Moreover, PDT-treated mice showed shrinkage of tumour size after 16 days, these results were confirmed by immunohistochemistry staining for the gross morphology of the engrafted tumour in brain slices.

**Figure 10:**
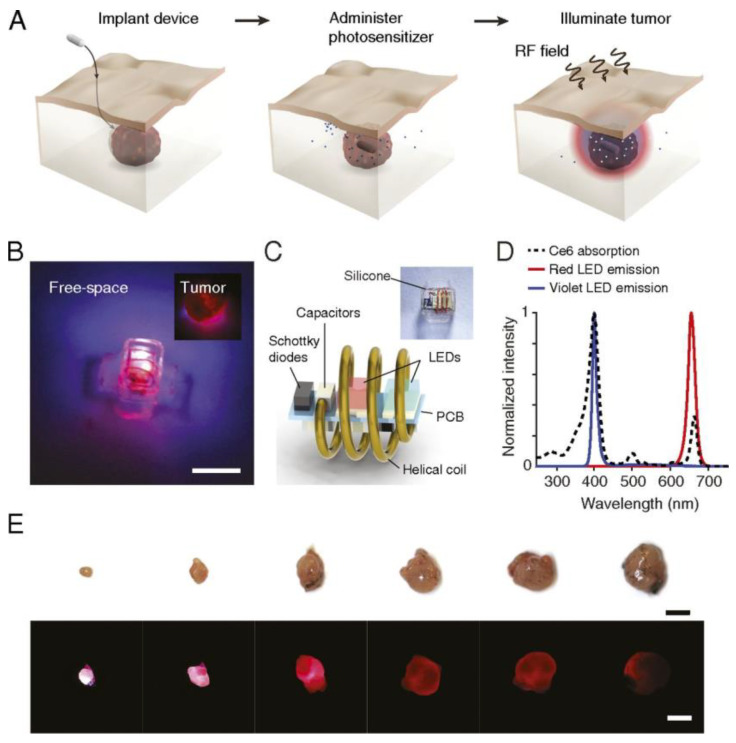
Wireless photonic PDT. (**A**) Schematic of the therapy. The therapy consists of implantation of wireless LED near the target region, administration of the PS, and wireless powering of the device. Light emitted by the device locally activates the PSs and induces tumour damage. (**B**) Image of the wireless photonic device emitting light. (Inset) The device illuminating an explanted tumour. (scale bar, 2 mm.) (**C**) Schematic of the device and individual electronic components. (**D**) The optical emission spectrum of the device from two LEDs. The emission wavelengths are centred at 400 nm (violet) and 660 nm (red), overlapping with the absorption peaks in the Ce6 spectrum. (**E**) Image panel of the penetration of light emitted by the device (radiant power, 1.3 mW) through tumours of increasing volume (scale bar, 5 mm). Reproduced from A. Bansal et al. PNAS; published by National Academy of Sciences under CC BY-NC-ND or CC BY license, 2018 [239].

Despite the promising results of the previous systems, most of them are still limited by the penetration depth of NIR radiation (< 2 cm) for external activation of the implant. To allow deeper penetration of external activation sources, other approaches have proposed the use of RF. We found the first proposal using RF in 2018 [239]. As can be observed in Figure 10, an implantable device (30 mg weight, 15 mm^3^) is placed inside the tumour. After 4 h of PS (chlorin e6, Ce6) administration, an RF signal (1–1.5 GHz) is applied to activate the LED module (encapsulated in PDMS). The device consists of a printed circuit board (PCB) connected to a helical coil that receives the incident energy of the RF. The PCB integrates the two LEDs (660 and 400 nm to match absorption peaks of Ce6) and the RF rectifier (capacitors and Schottky diodes). For the in-vivo test, a murine model of bladder carcinoma is used. Light power of 1.3 mW was applied for 30 min (2 J/cm^2^) and the second round of treatment was administered after 7 days. The results reveal a tumour volume reduction over time and in some cases a complete regression.

In 2019 Yamagishi et al. designed and manufactured a tissue-adhesive and near-field communication-based chip (a resonance frequency of 13.56 MHz and transmission power of 3 W), with red, green and blue LEDs, for mPDT [240]. The adhesive provides mechanical stability to the optical device tackling a major challenge on the use of implants in-vivo. The implants consisted of a commercial micro LED light source with an integrated coil (chips from Kyoritsu Electronic Industry) that has been encapsulated in PDMS and sandwiched in a bioadhesive material polydopamine (PDA). The PDA bounds to the biological tissue surface via a chemical reaction [240]. The approach is characterised by a low fluence rate µW/cm^2^ range and long-term treatment, allowing continuous delivery of light to the target tumours for at least 10 days [240]. The device led to significant antitumour effects with 1000-fold lower intensity than conventional PDT approaches. Because of that, the risk of thermal tissue damage is negligible. The same implant has been demonstrated recently using only green light (532 nm, power of 50–60 µW) and PS ALA [241]. The treatments with this implant required repeated PS administration. In the former study, intravenous administration of Phorifrin was carried out. In the latter, ALA was chosen because it can be introduced orally, thus partially reducing the burden to patients.

One of the main problems of RF activated systems is that micro-coils suffer from low power transfer efficiency and short transmission range, due to the small dimensions and the requirement of a precise alignment with the RF source [248]. To overcome these drawbacks, ultrasonic powering offers unique advantages such as higher power transfer efficiency for millimetre receivers, misalignment insensitivity, and a larger penetration depth (>20 cm) for implant activation [249]. As an example, in [242] an ultrasonically powered implant (~2 mm^3^) based on a piezoelectric transducer with surface-mounted red and blue LEDs (0.048 to 6.5 mW/cm^2^ for an ultrasound power of 185 mW/cm^2^ at 720 kHz) was proposed and demonstrated.

**Figure 11:**
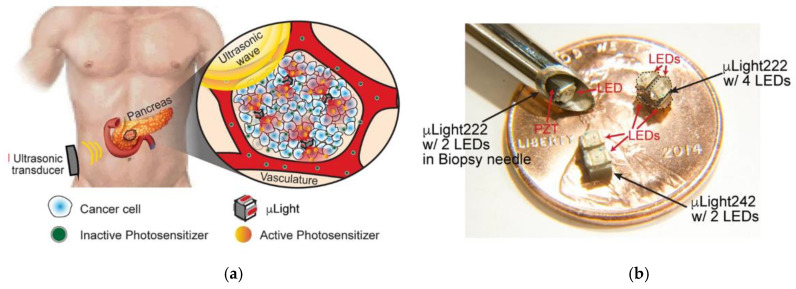
(**a**) Illustration of implantable µLight in a deep-seated tumour: ultrasonic waves applied from an external transducer travel through the tissue to trigger light generation by the μLight, thus activating a pre-delivered PS, which in turn generates ROS to kill cancer cells. (**b**) Photographs of fabricated prototypes. Reproduced with permission from A. Kim et al., Scientific Reports; published by Springer Nature, 2019 [242].

As can be observed in Figure 11a, the microlight sources can be implanted in a deep-seated tumour (e.g., pancreas). Multiple sources can be implanted at the same tumour due to misalignment insensitivity. The acoustic signal energizes the onboard LEDs, activating the PS. In Figure 11b, it is shown how the devices can be easily inserted with a biopsy needle (gauge 8). In vitro and in vivo test were made, in the latter case, the μLight242 was implanted in 16 mice with tumours of 200 mm^3^.

The treatment time was set to 30 min (2 J/cm^2^ light dose). A decrease in average tumour size of 20% was observed on day 8 [242]. This study demonstrates the feasibility and advantages of ultrasonically activated implant, opening a new way to produce PDT and mPDT.

To summarize this section, the main characteristics of implants described in this section are depicted in Table 5.

## 3. Discussion

A critical review of the light-based technologies and their influence on the PDT outcome has been addressed from superficial to deep techniques. The focus was set on the role of light source properties and parameters on the PDT efficacy and the light penetration into the tissue.

In particular, wavelength, coherence, incoherence, beam size, and the use of pulsed or CW illumination, are some of the factors covered for superficial and interstitial applications. PDT effect has been accomplished disregarding the light source nature, i.e., coherent or incoherent. There have been just a few attempts for direct comparison between light sources exhibiting similar results. Based on the existing literature, it is not possible to determine which light source type is more favourable for PDT or light penetration. However, it has been demonstrated that coherence is not lost within the tissue (<2 cm) by the observation of laser speckles in highly scattering media. Such speckles may exhibit significant intensity variations from the mean (up to 5 times). A possible implication of such variations on PDT is the local achievement of intensity thresholds for PS activation, thus deeper light penetration into additional regions that can experience PDT effects. Nevertheless, the relevance of such effects in clinical settings has not been determined. Specifically, to what extent is coherence influenced by the PS distribution and optical interaction has not been explored yet. Since achieving PDT of certain efficacy is also possible using incoherent sources such as LEDs, these light sources have strong potential for easier transfer to clinical settings offering more affordable solutions. One challenge is to extend beyond superficial applications and treat deep and large-seated tumours interstitially. Proper thermal control and power required (once coupled to a fibre) to surpass the minimum threshold for PS activation have to be demonstrated.

Regarding the properties of the incident light, such as pulsed or CW illumination, both regimes have shown beneficial effects. Most studies use CW light. Despite this, pulsed light has demonstrated some advantages as tissue re-oxygenation and re-accumulation of specific PS at the lesion. For light penetration, the use of pulsed light has shown a therapeutic threshold for PS activation in deeper regions of the tissue (with the same average fluence as CW but using higher fluence peaks). However, it is difficult to compare this type of illuminations based on existing literature given the diverse parameters employed—i.e., different PS, pulse duration and frequency, fluence rate and time, etc. Hence, some of the modest benefits of using pulsed or CW only apply under certain conditions. For this reason, the real advantages of pulsed illumination over CW are still debated. A commonly identified point is pulsed light typically favours apoptosis whereas CW favours necrosis. There has not been evidence contrary to tissue re-oxygenation partially avoiding hypoxia enabled by pulsed light. Systematic studies considering the implications of the pulse duration (and waveform) on the full dynamics of dependent parameters (PS activation, concentration, oxygen conditions) might shed light on the effects of using pulsed light. Other spatially-tuned light waveforms are deemed highly attractive in order to inhibit scattering and achieve deeper light penetration. Their suitability has to be envisaged.

Finding which type of light and beam parameters are better for PDT also benefits other advanced techniques (e.g., implants), in which dedicated circuits (or modes of activation) can be implemented. The main differentiators of I-PDT are the treatment of deep-seated tumours (but completely localized) and the possibility to perform full dosimetry not easy to implement by other PDT modalities.

For deep PDT, several techniques have been demonstrated. We have addressed the most relevant: chemiluminescent, bioluminescent sources, NIR, X-rays or Cherenkov radiation, and implants. Table 6 summarizes the main features and challenges of these techniques.

In the case of NIR radiation, one photon excitation is limited by the penetration depth and the available PS. TPA and FRET mechanisms are compatible with the use of longer excitation wavelengths and increase light penetration depth. For TPA, fs-laser pulses and diffraction-limited beam sizes are employed, enabling the treatment of highly localized regions (and possibly sectioning in the axial direction) and opening the possibility to avoid critical organs. It is worth mentioning that high selective treatments (disregarding sectioning) are not a particular advantage of TPA, they can also be achieved by one-photon excitation systems, but sacrificing penetration depth. To what extent the penetration depth of these focused beams is limited by the beam size (as identified in Section 2.2.2) is unknown. The low cytotoxic effects and low absorption cross-sections of the available PSs for TPA limit their use. Novel nonlinear upconversion techniques have emerged, offering better treatments as compared to TPA alone and using more conventional PSs. Their use might be limited to fine treatments due to the very small beams in which practical implementation for large tumours seems unfeasible. UCNPs (PS activation driven mainly by FRET) enable the excitation of common PSs at the visible region by converting NIR radiation to a wide range of visible light wavelengths. One of the main challenges using UCNPs is the lack of understanding of PDT mechanisms. Toxicity to normal cells has been reported, indicating that UCNPs can produce effects by themselves, and possibly, not only related to PDT mechanisms. More studies to elucidate the mechanisms of action are required to further understand the real effect of UCNPs.

Cherenkov and X-rays sources offer unlimited penetration. Despite this is a great advantage, both sources rely on ionising radiation that can damage the DNA. As it happens with UCNPs, SCNPs for ionising radiation have shown biocompatibility issues whose mechanisms are not clear. In the case of Cherenkov radiation, there is no explanation for how such small light doses can produce such a PDT effect. Further research is required to shed light on the intriguing results and to evaluate the realistic potential of these techniques.

Self-illuminated systems have demonstrated a therapeutic effect. However, their ability to translate to the clinic is in doubt. In the case of CL systems, the mechanisms of excitation are not fully understood, as only PDT effects are evaluated and usually based merely on tumour size observations. For BRET, there is also uncertainty in light fluences in tissue and the PDT effect. It is highly complex to determine the efficiency of each energy conversion step involved in the process of self-illumination. Besides, the required short distance between the substrate, the catalyst and the PS is not easily accomplished, and significant variability of the proximity may be found in real systems. Overall, CRET and BRET systems have unlimited penetration depth and no external source is required, but they are at a very early stage of development. If some issues—such as biocompatibility, uncertain dose, and specificity—are addressed, they have strong potential to become a magic bullet against cancer.

Concerning the emerging technological developments of implants, nowadays it is possible to design biocompatible implants (encapsulated with transparent polymeric materials) of different types, shapes and sizes (mm-range or below) that could be strategically allocated on the tumour surface to achieve less invasive mPDT. Tissue-adhesive implants have shown that movement difficulties and stability of the optical devices can be minimized [240]. Implants are advantageous over cases requiring bulky systems (i.e., optical fibre setups), in which the movement restriction turns into less practical treatments. Besides, implants could provide access to locations where interstitial delivery devices (see Section 2.2.4) have limitations. Despite all of these advantages, implants can be difficult to allocate properly for repeating the treatment in growing tumour. In addition, the risk of seeding cancer cells has to be considered for insertion and removal of the implants.

Examples of light sources externally activated are persistent luminescent materials—or so-called “optical batteries”—LED sources and even upconversion materials. Specifically, PLNPs can emit light from minutes to hours and for several wavelengths (from UV to NIR). However, most of the PLNPs utilise Cr^3+^ ions, and although they are mixed with an encapsulation material the heavy toxicity of Cr^3+^ ions would not meet requirements for environmental, biocompatible, and biosafety aspects. Whereas LEDs offer great flexibility in wavelength choice, power, and modes of activation, the implant geometry is limited by the size of the coil or receiver for activation. Another alternative is the use of UCNPs, which assist in the challenges of UCNPs (not implanted) regarding its retention in the central nervous system and toxicity avoidance by encapsulating the materials within the implant.

Considering the modes of external activation of the implants, different radiation sources (e.g., NIR radiation, RF, NFC, ultrasounds) have been demonstrated. Each radiation source has its particularities and specific benefits and weaknesses depending on the application (Table 4). For instance, ultrasonic radiation offers the highest penetration into the tissue for external activation of the implants (>10 cm), as compared to RF (<10 cm) and NIR (<2 cm). Whereas RF requires precise alignment between the energy radiation source and the receiver in the implant, ultrasonic radiation does not require alignment facilitating the allocation of multiple implants on the tumour surface. The uniformity on the activation and irradiation of multiple implants is still unknown, and thus their implications on PDT. NIR has limited penetration depth, but allows the use of UCNPs with the benefits explained above.

Different PSs have also been tested (e.g., 5-ALA, HPPH, Rose Bengal, Ce6, verteporfin) with treatment times extended up to tenths of hours, all implants achieving anti-tumour effects. Most studies used animal models to demonstrate the capabilities of the specific implant designs. Some others are targeting the treatment of deep-seated tumours such as the pancreas, in which 80% of the tumours are unresectable and easily recurrent [242]. mPDT through implants can mitigate such effects and find application in other tumours. Implants including the PS (RB) have been reported. This approach may offer important benefits. The PS is highly localized within the implant; thus, in principle, PS infusion is no longer required, avoiding drug-light intervals and burden to patients. However, it is well-established that direct injection of PS into the tumour (as also done for UCNPs) hinders the shutdown of microvessels which is one of the main cell death mechanisms [90]. Moreover, mPDT requires several PS infusion sessions increasing the invasions if non-IV PS is uptaken. Another issue is that the singlet oxygen is generated inside the implant and the cytotoxic effect relies on the diffusion of the singlet oxygen to the tumour passing through the encapsulation (i.e., PDMS) to reach the tumour cells. It is known that the diffusion length of the singlet oxygen in cells is <1 µm (for typical lifetimes <100 µs) and up to 3 mm, considering a hypothetical lifetime of seconds. Hence, their effect might be superficial.

In spite that many light sources emit in the visible range with limited penetration depth within the tissue, one of the main differentiators of implants is the possibility to use mPDT, which demonstrated greater necrosis depth with beneficial effects over conventional PDT [81,210,240,244,245,246].

## 4. Conclusions

We have presented an overview of the status and challenges of the most relevant PDT modalities. The focus was set on the influence on PDT of light sources, devices, and systems. For conventional light sources mainly used for superficial and I-PDT (lasers, LEDs, broadband lamps), the questions about whether the nature of the light sources and parameters (coherent, incoherent, monochromatic, broadband, pulsed, CW) are more favourable for PDT remain unclear. The influence on PDT of one or another light source and beam parameters is highly dependent on the fluence, treatment site, oxygen, and the PS. We also identified the necessity to properly report the PDT dose and beam parameters used for the treatments for a proper comparison between results. Answering these questions is of paramount importance not only for conventional PDT, but also for other PDT modalities reviewed in this work, e.g., implants.

For some deep PDT modalities such as nonlinear optical techniques or ionising radiation, we identified that the main challenges or limitations are the development of enhanced PSs, non-toxic UCNPs, and the use of ionising radiation. Self-illuminated systems are considered a potential magic bullet for cancer. They are in an initial stage and, currently, few studies report weak self-luminescence, toxicity, and low specificity without a clear understanding of the limiting factors and mechanisms of action.

There is strong potential for emerging implants given the possibility to achieve mPDT without repeated invasions. There is a wide palette of implant designs with demonstrated PDT capabilities and weaknesses. Some offer the possibility to adapt to different treatment sites. Ultrasound-activated implants appear as an attractive implementation offering deep external activation and the possibility for multiple implants allocation. On the contrary, some designs including persistent luminescence materials feature insufficient doses and stability issues. Full dosimetry might be unfeasible, and partial dosimetry has to be envisaged.

Each PDT modality offers unique capabilities. Emerging technologies overcome some of the initial challenges, but in turn, pose limitations. There is not a magic bullet for cancer based on PDT. Presently, instead of offering a broad solution, each PDT modality may find specific niches or be used in combination with other PDT modalities or therapies. Undoubtedly, PDT effects are beneficial for many treatment sites, and emerging technologies are opening new avenues towards enhanced PDT.

## Figures and Tables

**Table 1:** Main benefits and limitations of coherent (laser) and non-coherent (LED, lamp) light sources in superficial PDT. The table is adapted from [8].

Light Source	Main Benefits	Limitations
Laser (coherent)	<0.1 nm spectral bandwidth High power Efficient coupling to optical fibres Uniform irradiance can be easily achieved Adaptive emission (VCSEL, Edge-emitting laser) Faster modulation than LEDs Possibility for ultra-short pulses (fs-regime)	Expensive High maintenance Bulkier than an LED Less choice of wavelengths
LED (non-coherent)	Low cost Small Adaptive emission (SLED, ELED) Used for whole-body or point treatment LEDs can fit down biopsy channels permitting deep-seated PDT	5–10 nm spectral bandwidth (FWHM) Large beam divergence Thermal effects for I-PDT (low electro-optical conversion efficiency)
Lamp (non-coherent)	Low cost Simple design Wide illumination field Multi-wavelength irradiance	UV and NIR radiation (optical filtering is needed) Large beam divergence High coupling losses with light guides

**Table 2:** Characteristics and conclusions derived from empirical studies using pulsed light sources in superficial and I-PDT. *T_ON_* stands for the pulse duration of the laser, PRR for the pulse repetition rate or frequency, *φ* for the average fluence rate and *Φ* for the average fluence. Adapted and updated from [97].

	Pulsed Light Source	Parameters	CW Light Source	Conclusions
[102,103]	Long-pulsed dye laser	*λ* = 585 nm *T_ON_* = 1.5–40 ms PRR = 1 Hz *Φ* = 4–7.5 J/cm^2^	NA	Treatment of AKs using pulsed lasers following topical 5-ALA application is safe and effective.
[101]	Pulsed Dye Laser Broadband flashlamp filtered pulsed light	*λ* = 595 nm *T_ON_* = 10 ms *Φ* = 7.5 J/cm^2^ *λ* = 500–650 nm *T_ON_* = 20 ms *Φ* = 24 J/cm^2^	CW blue light *φ* = 10 mW/cm^2^	Pulsed light sources are capable of activation of PDT but produce dramatically less PDT reaction than the standard CW blue-light source.
[94]	Nd:YAG laser pumped optical parametric oscillator (OPO)	*λ* = 690 nm *T_ON_ =* 10 ns PRR = 10 Hz *φ* = 300 mW/cm^2^	Argon-laser + Dye; *λ* = 690 nm *φ* as pulsed	Pulsed irradiation allowed modest deeper light penetration in tissue.
[100]	Nd:YAG laser-pumped OPO	*λ* = 514.5 nm *T_ON_* = 7–9 ns PRR = 10 Hz *φ* = 100 mW/cm^2^ *Φ* = 1 to 10 J/cm^2^	Argon-laser *λ* = 514.5 nm *φ*, *Φ* as pulsed	Dead cells irradiated by the pulsed laser light were induced through apoptosis. CW laser light irradiation led to necrosis.
[95]	Nd:YAG + OPO system	*λ* = 670 nm *T_ON_* = 5 ns PRR= 30 Hz *φ* = 180 or 270 mW/cm^2^ *Φ* = 40 J/cm^2^	Diode laser λ = 670 nm *φ*, *Φ* as pulsed	Pulsed light caused suppressed oxygen consumption and lower cytotoxicity Pulsed light induced a lower decomposition rate of the PS
[96]	Ti:sapphire + optical parametric amplifier	*λ* = 630 nm *T_ON_* = 70 fs PRR= 1 kHz *φ* = 74 mW/cm^2^ *Φ* = 150 J/cm^2^	Diode laser *λ* = 630 nm *φ*, *Φ* as pulsed	Twice as deep necrosis for ultra-fast pulsed laser using hematoporphyrin from Photogem^®^. Temperature increase <4 °C using a pulsed laser.
[99]	Diode pumped solid state yellow laser	λ = 584 nm *T_ON_* = 15 ns PRR = 10 Hz *φ* = 160 mW/cm^2^ *Φ* = 140, 170 and 200 J/cm^2^	Diode Laser λ = 593 nm *φ*, *Φ* as pulsed	Pulsed mode mainly led to apoptotic cell death, while, in the case of CW mode, the cancer cells underwent necrosis. Similar photobleaching observed for both light sources
[98]	Semiconductor laser	*λ* = 670 nm *T_ON_* = 200 ms Pulse repetition period = 700 ms *φ* = 2.5, 5, 10, 15 and 20 mW/cm^2^ *Φ* = 0.625 to 5 J/cm^2^	*λ* = 662 ±3 nm *φ*, *Φ* as pulsed	Pulsed promoted apoptotic cell death of k562 cells whereas CW induced necrotic cell death

**Table 3:** Excitation and emission wavelengths of some reported UCNP, and available laser sources. Note UCNPs excited with 975–980 nm match a high absorption peak from water (Figure 1) which may cause excessive heating of the tissue. To overcome this, neodymium ions can be added to the UCNP which absorb ~800 nm [153].

Excitation (NIR)	Emission (nm)	Ref.	Laser Diodes (max. Power)
980 nm	345, 360, 450, 475	[154,155,156,157]	L980P010 (10 mW)
	450, 475	[158]	LP980-SF15 (15 mW)
	540	[159,160]	L980P030 (30 mW)
	520, 545, 660	[161]	L980P100A (100 mW)
	409, 541, 656	[162]	L980P200 (200 mW)
	660	[162,163,164,165,166,167,168,169]	C3-980-0500-S50 (500 mW)
	540, 660	[170,171,172]	WSLD-980-001-2 (1 W)
975 nm	340, 360, 445, 475 620 660	[173] [174]	0975L-14A-NI-PT-NF (70 mW)
			RLTMDL-975-100 (100 mW)
			RLTMDL-975R-300 (300 mW)
			PL980P330J (330 mW)
			RLTMDL-975-500 (500 mW)
			RLTMDL-975-1W (1 W)
808 nm	345, 360, 450, 475 350, 450 540 540, 660 543, 654 660	[175] [156] [176] [175] [177] [178]	L808P010 (10 mW)
			L808P030 (30 mW)
			DBR808PN (42 mW)
			LP808-SA60 (60 mW)
			M9-808-0150 (150 mW)
			L808P200 (200 mW)
			FPL808S (250 mW)
			LD808-SE500g (500 mW)
			L808P1000MM (1 W)

**Table 4:** Some reported X-ray SCNPs with the required energy to activate them and the emission wavelengths. Adapted with permission from [179].

Excitation (X-ray Dose)	Emission (nm)	X-ray Scintillator (Size)	Ref.
6 MeV, 30 keV 1–6 Gy	340	CeF_3_ (9 nm)	[188]
50 keV 1–10 Gy	520	SrAl2O_4_:Eu^2+^ (407 nm)	[189]
90 keV, 3 Gy	520	LaF_3_:Ce^3+^ (2 µm)	[190]
75 keV	544	LaF_3_:Tb (40 nm)	[191]
75 keV	540	LaF_3_:Tb silica coated (45 nm)	[192]
6 MeV, 0.4–2 Gy	545	SiC/SiOx core/shell nanowires (40 nm)	[193]
80 keV	540	LaF_3_:Tb (25 nm)	[194]
44 keV, 11 Gy	540	Tb_2_O_3_ coated polysiloxane (10 nm)	[195]
15 keV	595	GdEuC12 (4.6 nm)	[196]
225 keV, 2 Gy	500	HfnMOL (1.2 nm)	[197]
120 keV, 2 Gy	510	ZnS:Cu,co (4 nm)	[198]
220 keV, 8 Gy	305	LiYF_4_:Ce (35 nm)	[199]
50 keV, 5Gy	720	LiGa_5_O_8_:Cr (100 nm)	[200]
160 keV, 5 Gy	543	NaLuF_4_:Gd,Eu (25 nm)	[201]
1.48 keV	300–450	Y_2.99_Pr_0.01_Al_5_O_12_@SiO_2_ (75 nm)	[202]

**Table 5:** Description of representative implants used for mPDT.

Light Source	Emission	Implant Size	Encapsulation	External Source (Activation/Charging)	PS
(ZGC) PLNPs [236]	695 nm Hours of emission	-	PLGA/NMP oleosol	LED: 400–750 nm for 2 or 5 min	HPPH, Photoclor Abs. peaks: 385 nm, 666 nm Emission: 670 nm
GPM (+PS) [237]	520 nm >2 h emission	Various shapes and sizes (mm-range)	PDMS	Laser: 980 nm 2 W/cm^2^ for 5 s	Rose Bengal Abs. peak: 559 nm Emission: 571.5 nm
UCNPs [238]	635 nm	3 cm	PEGDA+FEP	Laser: 980 nm 1109 mW/cm^2^ for 5–10 min	5-ALA Abs. Peaks: 405 nm, 630 nm
LEDs [239]	660 and 440 nm	15 mm^3^	medical-grade silicone	RF (1 and 1.5 GHz) for 30 min	Ce6 Abs. Peaks: 400 nm, 663 nm Emission at 667 nm
LEDs [240]	630, 530, 460 nm	LED chip: 7.0 × 11 × 0.8 mm^3^ +PDMS+PDA: ~650 nm-thick	PMDS+PDA (adhesive)	Near-field communication (13.56 MHz) for 10 days	Photofrin-saline & 5-ALA
LEDs [242]	640, 470 nm	2 × 2 × 2 mm^3^ 2 × 4 × 2 mm^3^ +surface mounted LEDs	5 μm of parylene-C	Ultrasonic 185 mW/cm^2^ at 720 kHz for 30 min	Verteporfin Abs. peak: 415, 580, 680 nm Emission: 690 nm

**Table 6:** Main features and challenges of deep PDT modalities and sources. Only TPA uses direct excitation of the PS whereas all the other deep PDT modalities excite the PS indirectly.

Deep PDT Modality	Source	Main Potential and Benefits	Main Challenges and Limitations
NIR radiation	TPA (ps-fs lasers)	High selectivity PSs more suitable for imaging-guided PDT	Not easily accessible systems Low ROS yield for TPA PSs Low absorption cross-sections for TPA PSs Heat-Damage by fs-laser Not suitable for large tumours
NIR radiation	TPA, CARS, FWM, SHG. (ps-fs lasers)	High selectivity Deeper penetration and enhanced PDT effects than TPA alone Common PSs may be used	Not easily accessible systems Heat-damage by fs-laser Not suitable for large tumours
NIR radiation	UCNPs	Conventional PSs excited in Soret band Lower power than TPA CW can be used	Retention in the central nervous system Toxicity in normal cells Bioclearance For FRET activated PS (most efficient), it requires proximity of UCNPs and PS (1–10 nm) Relatively low quantum yield
Ionising radiation	X-rays	Unlimited depth penetration	Ionising radiation Biocompatibility issues of some SCNPs.
Ionising radiation	Cherenkov	Unlimited penetration depth Easier combination with radiotherapy No external radiation	Ionising radiation or radioisotopes required Understanding of PDT mechanisms is needed Weak luminescence
CRET, BRET	FireFly Renilla	Unlimited penetration depth No external radiation	Few studies, contradictory results Not clear mechanisms of action Low specificity Toxicity in normal cells Intermediate steps decrease efficiency Weak self-luminescence
Implants	-	Valid for mPDT Repeated PDT with no invasion Low fluence rates, no thermal damage. Possibility for multiple implants allocation LEDs offer higher fluence rates than other sources for implants LEDs can target several PS abs. peaks	mPDT requires repeated PS infusion Perform dosimetry Compatibility with dosimetry tools Stability of the implant Placement difficulty Undesirable interaction with tumour giving rise to possible seeding of cancer cells.
Implants (NIR)	PLNPs	Injectable within tumour Luminescence from minutes to hours UV to NIR radiation	Poor tumour retention Placement control or targeting Insufficient doses Biocompatibility and biosafety validation required Limited penetration depth of NIR
Implants (NIR)	GPM (+PS+upconversion materials)	Flexibility in shape and size design Localized PS in the implant No clearance of PS required No IV injection of upconversion materials	Hinder cell death mechanisms because intratumourally injection of PS is not optimum PDT relies on singlet diffusion in the cells PS re-infusion required for mPDT difficult High power for activation Limited penetration depth of NIR
Implants (NIR)	UCNPs	No diffusion of UNCPs Allows to remove UCNPs Avoids potential UNCPs toxicity No electronics, no battery charging Bendable light guides	Limited penetration depth of NIR Constant activation of UCNPs
Implants (RF-NFC)	LEDs	Stability demonstrated by tissue-adhesives Penetration depth <10 cm for external activation	Difficult alignment and low coupling efficiency of source-receiver Limited size and shapes due to coil or antenna required and electronics
Implants (US)	LEDs	Penetration depth >10 cm for external activation Multiple implants can be activated	Uniformity of treatment when using multiple implants Increased complexity in dose determination when using multiple implants
